# Disentangling Thermal from Electronic Contributions in the Spectral Response of Photoexcited Perovskite Materials

**DOI:** 10.1021/jacs.3c12832

**Published:** 2024-02-15

**Authors:** Lijie Wang, Razan Nughays, Thomas C. Rossi, Malte Oppermann, Wojciech Ogieglo, Tieyuan Bian, Chun-Hua Shih, Tzung-Fang Guo, Ingo Pinnau, Jun Yin, Osman M. Bakr, Omar F. Mohammed, Majed Chergui

**Affiliations:** † Laboratory of Ultrafast Spectroscopy, ISIC and Lausanne Centre for Ultrafast Science (LACUS), École Polytechnique Fédérale de Lausanne (EPFL), Lausanne CH-1015, Switzerland; ‡ Advanced Membranes and Porous Materials Center (AMPM), Division of Physical Science and Engineering, King Abdullah University of Science and Technology, Thuwal 23955-6900, Kingdom of Saudi Arabia; § Department of Applied Physics, The Hong Kong Polytechnic University, Kowloon 999077, Hong Kong, P. R. China; ∥ Department of Photonics, National Cheng Kung University, Tainan 701, Taiwan ROC; ⊥ KAUST Catalysis Center, Division of Physical Sciences and Engineering, King Abdullah University of Science and Technology, Thuwal 23955-6900, Kingdom of Saudi Arabia; ▲ Department of Chemistry, University of Basel, Klingelbergstrasse 80, CH-4056 Basel, Switzerland

## Abstract

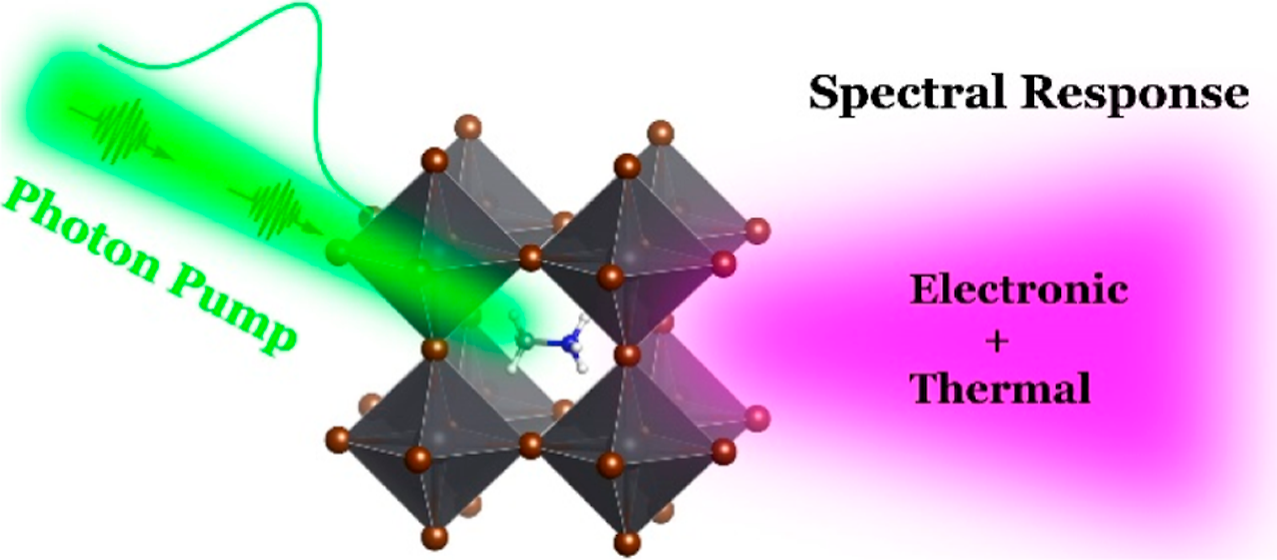

Disentangling electronic and thermal effects in photoexcited perovskite materials is crucial for photovoltaic and optoelectronic applications but remains a challenge due to their intertwined nature in both the time and energy domains. In this study, we employed temperature-dependent variable-angle spectroscopic ellipsometry, density functional theory calculations, and broadband transient absorption spectroscopy spanning the visible to mid-to-deep-ultraviolet (UV) ranges on MAPbBr_3_ thin films. The use of deep-UV detection opens a new spectral window that enables the exploration of high-energy excitations at various symmetry points within the Brillouin zone, facilitating an understanding of the ultrafast responses of the UV bands and the underlying mechanisms governing them. Our investigation reveals that the photoinduced spectral features remarkably resemble those generated by pure lattice heating, and we disentangle the relative thermal and electronic contributions and their evolutions at different delay times using combinations of decay-associated spectra and temperature-induced differential absorption. The results demonstrate that the photoinduced transients possess a significant thermal origin and cannot be attributed solely to electronic effects. Following photoexcitation, as carriers (electrons and holes) transfer their energy to the lattice, the thermal contribution increases from ∼15% at 1 ps to ∼55% at 500 ps and subsequently decreases to ∼35–50% at 1 ns. These findings elucidate the intricate energy exchange between charge carriers and the lattice in photoexcited perovskite materials and provide insights into the limited utilization efficiency of photogenerated charge carriers.

## 1 Introduction

Time-resolved pump–probe spectroscopy has revolutionized the investigation of photoinduced generation and evolution of electron–hole pairs in materials on the femtosecond (fs) to picosecond (ps) time scales.^1−3^ Probe ranges from the infrared (IR) to the visible region,^4−8^ and recently into the deep-ultraviolet (UV) region,^9−12^ have been used. The photoinduced transients of solid materials arise from multiple electronic and thermal effects,^13−15^ which can be difficult to disentangle. Lattice heating via carrier–phonon coupling produces strong interplay between photoinduced electronic responses and thermally induced changes in optical properties, even at very low fluences,^16,17^ and this interplay also evolves with time. In many instances, the analysis of photoinduced transients and dynamics has predominantly focused on electronic processes, neglecting the accompanying spectral response originating from heating, especially in semiconductor solar materials.^18−21^

Among these, perovskites are regarded as some of the most promising materials due to their distinctive combination of strong photoabsorption, long charge carrier diffusion lengths, and low-cost fabrication processes.^22−24^ One prominent example is the hybrid organic–inorganic perovskite, exemplified by methylammonium lead bromine (MAPbBr_3_). The key metric for their applications is the photoelectronic conversion efficiency. However, the photogenerated electrons and holes partly lose their energy by so-called phonon emission via carrier–phonon coupling at an ultrafast time scale,^25,26^ thereby limiting the actual utilization efficiency of charge carriers. More importantly, the consequent lattice heating effects can alter the intrinsic optical characteristics of the material and complicate their transient spectral responses,^27−30^ making it challenging to resolve and quantify the electronic and thermal contributions in time-resolved signals, not to mention their temporal evolutions.

**Scheme 1:**
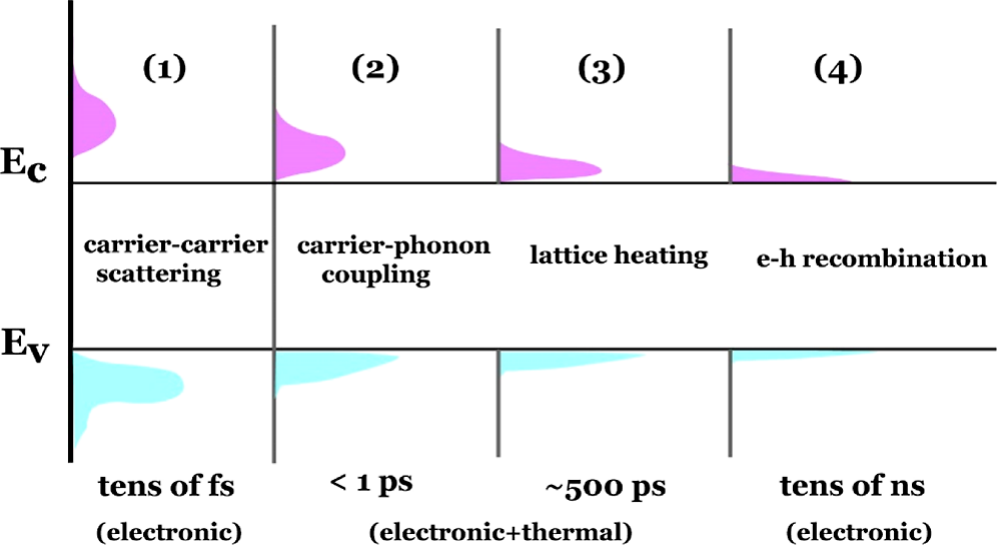
Carrier Cooling Processes and Their Corresponding Electronic and Thermal Contributions in the Spectral Response In process (1), carrier–carrier scattering occurs immediately after carrier generation, leading to a predominantly electronic TA signal. Subsequently, the thermal response emerges with carrier–phonon coupling, initiating the growth in processes (2) and (3) until ∼500 ps. Afterward, the spectral thermal response diminishes and fades away within a few nanoseconds, while the TA signal is dominated by electronic contributions lasting tens to hundreds of nanoseconds.

In this study, we conducted broadband transient absorption (TA) experiments in the visible and mid-to-deep-UV regions on a thin film of the MAPbBr_3_ perovskite to investigate the ultrafast processes of energy transfer from photoexcited charge carriers to phonons (lattice) in different low- and high-energy bands. A deep-UV probe accesses the high-energy excitations, offering a significant advantage compared to conventional visible to terahertz probes.^12,31−33^ Furthermore, this region is less influenced by the signal resulting from free carrier absorption.^34^ The photoinduced data were analyzed using a global lifetime analysis (GLA) method to obtain decay-associated spectra (DASs),^35^ which offer a compact representation of the kinetic information associated with specific lifetimes. In order to disentangle the electronic from the thermal effects, we recorded the temperature-dependent spectra using in situ variable angle spectroscopic ellipsometry (VASE) across a wide spectral range, and we compared the derived difference spectra to the photoexcited TA ones (Figure S1). Some striking similarities arise between thermal- and photoinduced responses at intermediate delay times. In order to weigh the relative contributions of thermal versus electronic effects and their evolution over time, we reconstructed the TA spectra from 1 ps time delay onwards, using combinations of DASs and temperature-induced differential absorption (TDA) spectra. Our findings demonstrate that the thermal contribution exhibits an increase by ∼55% in ∼250–400 ps and then a decrease to ∼40% by ∼1 ns (see Scheme 1 for the sequence of carrier cooling processes after photoexcitation and their corresponding electronic and thermal contributions in the spectral response). This behavior is related to the lattice heating by carrier–phonon energy exchange, which increases at early times and then decreases as cooling evolves in tens to hundreds of ps. The experimental procedures are described in the Supporting Information.

## 2 Results

### 2.1 Steady-State Spectroscopy

The room temperature steady-state absorption of a MAPbBr_3_ single crystal was extracted by fitting the ellipsometry spectra measured at three different angles (65, 70, and 75°). As depicted in Figure S2, a distinct excitonic feature appears at ∼2.3 eV, along with multiple absorption peaks at approximately 3.4, 3.8, and 4.45 eV. These features can be attributed to specific transitions according to the calculated band structure diagram shown in Figure S3 and are annotated in the absorption spectrum (see also Note S1, Supporting Information). They are identified as VB1 → CB1 at the R point, VB3 → CB1 at the R point, VB1 → CB1 at the M point, and VB1 → CB1 at the X point, respectively.^36,37^ Density functional theory (DFT) calculations were performed using the generalized gradient approximation (GGA) and Perdew–Burke–Ernzerhof (PBE) functionals. In the band structure diagram, the excitation photon energy used for both the visible and mid-to-deep-UV probes was fixed at 3.1 eV (400 nm) and is denoted by the blue arrow, while the probed signals detectable by the broadband visible and mid-to-deep-UV probes are represented by the yellow and red arrows, respectively.

### 2.2 Visible Transient Absorption Spectroscopy

**Figure 1:**
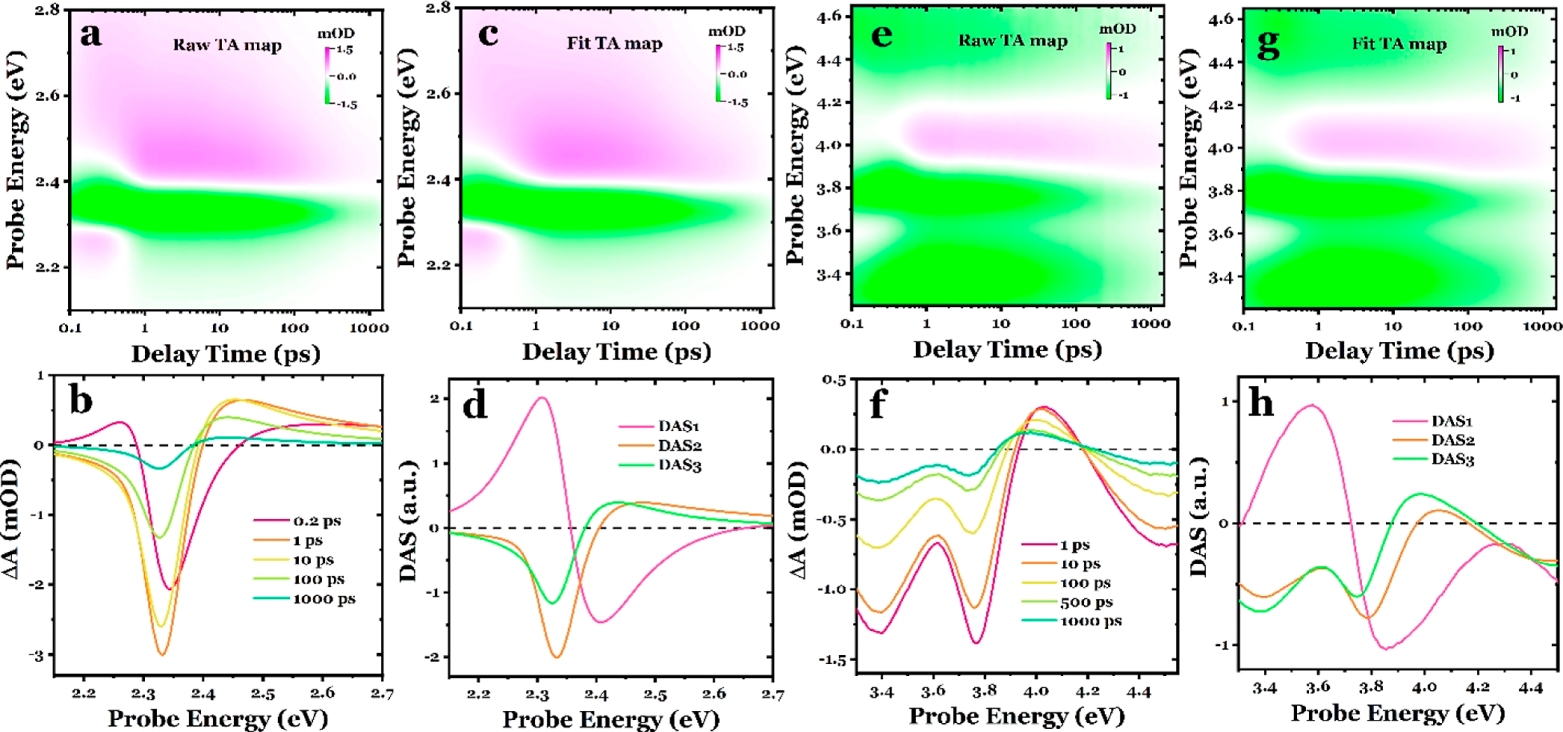
Photoinduced transient responses in the MAPbBr_3_ perovskite probed in the visible and UV spectral regions. (a) Experimental TA time–energy map probed in the visible spectral region. (b) Corresponding TA spectral traces at 0.2, 1, 10, 100, and 1000 ps, respectively. (c) Fitted TA map obtained using global lifetime analysis. (d) DASs corresponding to the initial (subps), mid (∼45 ps), and long-term (∼740 ps) evolution processes. (e) Experimental TA time–energy map probed in the mid-to-deep-UV spectral region. (f) Corresponding TA spectral traces at 1, 10, 100, 500, and 1000 ps, respectively. (g) Fitted TA map obtained using global lifetime analysis. (h) DASs corresponding to the initial (subps), mid (∼35 ps), and long-term (∼800 ps) evolution processes.

The MAPbBr_3_ thin films were first probed in the visible spectral region as a benchmark experiment, and the results are consistent with reported transient responses.^38−45^Figure 1a shows the time–energy TA map of the MAPbBr_3_ perovskite under 3.10 eV excitation, and Figure 1b shows the corresponding spectral traces at different time delays. The typical negative signal at the optical band gap (BG) around ∼2.35 eV and a broad, weak absorption signal on the higher-energy side show up. Also, a weak positive signal promptly appears at subpicosecond time scales on the lower-energy side of the optical gap, which is attributed to band gap renormalization (BGR). The origin of these transient signals in the visible region has been extensively discussed previously.^38,39,46−48^ It is worth noting that the negative peak at 2.35 eV undergoes a red shift at early times, which can be attributed to the interplay between the red shift due to BGR and the blue shift resulting from the Burstein–Moss effect (see Figures S4 and S5 for the normalized spectral traces and time traces, Supporting Information).^49^ However, it has been reported that under band edge excitation, inorganic perovskite nanocrystals induce a transient blue shift attributed to polaron formation on the 300 fs time scale.^50,51^

Given the complexity of the TA signal composition, it is necessary to perform a global analysis that simultaneously examines multiple kinetic traces recorded at different probe energies, enabling the extraction of different signal components at specific lifetimes. The global lifetime analysis (GLA) was performed using a discrete sum-of-exponentials function^35^
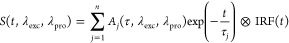
1where the “τ”s represent the global lifetimes and the “*A*”s are the amplitudes for each kinetic trace. The detected signals are convoluted with the instrument response function (IRF), which is modeled by a polynomial function^52^
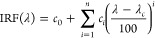
2

The time zero position at the central wavelength, λ_c_, is given by *c*_0_. GLA results in the so-called DAS, where the pre-exponential amplitudes for each lifetime component are plotted as a function of the probe wavelength, λ_pro_. It is worth noting that at very early delay times (sub-ps time scale), the spectral red shift at the BG transition could yield an orthogonal component resembling a derivative-like shape, which serves as evidence for the BGR effect at the shortest time scales.^53,54^ In the mid-to-long decay times, spectral shifting primarily arises from the spectral overlap, influencing the relative spectral weight of bands; nevertheless, this phenomenon does not impact the outcomes of the GLA; in fact, the GLA can effectively separate the overlapped spectral contributions, as demonstrated later. Figure 1c displays the TA map retrieved from the extracted DAS. They successfully capture all of the spectral features observed in Figure 1a. The residuals are presented in Figure S6. The resulting DASs are associated with three different lifetimes (Figure 1d) corresponding to the initial (subpicosecond), mid (∼45 ps), and long-term (∼740 ps) evolution processes. DAS1 represents the spectrum for the sub-ps lifetime component. It exhibits a distinctive derivative-like shape that is positive on the low-energy side and negative on the high-energy side, providing a clear indication of BGR and, therefore, a pure electronic response. Yet, accurately determining the early time scale of BGR necessitates a more sophisticated lifetime density distribution analysis, which is beyond the scope of this study. DAS2 and DAS3, corresponding to longer lifetimes, exhibit rather similar spectral profiles, and are analogous with the TA spectral trace observed at longer delay times.

### 2.3 Mid-to-Deep-UV Transient Absorption Spectroscopy

Figure 1e shows the time–energy TA map of the MAPbBr_3_ perovskite probed in the UV spectral region, recorded under pump conditions identical with those of the visible probe experiments. The corresponding spectral traces are shown in Figure 1f. The transients exhibit three pronounced negative bands at approximately 3.4, 3.8, and 4.45 eV, accompanied by a positive signal at around 4.15 eV. Just as in the visible range (Figure 1a), these signals arise promptly and thereafter evolve differently and persist up to 1 ns, except for the 4.15 eV feature, which shows an approximately 500 fs delayed rise (Figure S7). Additionally, this feature shifts to lower energies by ∼85 meV from 1 to 1000 ps. Moreover, the ratio of the negative bands at ∼3.4 and ∼3.8 eV varies with delay, with the dominance of the ∼3.8 eV band decreasing at later delay times. According to the band assignments described in Figures S2 and S3, the features observed at approximately 3.4, 3.8, and 4.45 eV coincide well with the interband transitions at different R, M, and X symmetry points, while the photoinduced absorption signal at ∼4.15 eV could result from symmetry breaking.^55^ Given that the excitation photon energy falls below the edge energy of the high-energy bands at the M and X points but exceeds the energy gap at the R point, the negative signals observed at ∼3.8 eV (M point) and ∼4.45 eV (X point) exhibit distinct signal rising behaviors compared to those at ∼3.4 eV (R point), as illustrated in Figure S8. These disparities suggest the involvement of different mechanisms in the generation of the TA signal, e.g., the negative signal at ∼3.4 eV can be attributed to the accumulation of excess electrons at the bottom of the CB at the R point, while the signals at ∼3.8 and ∼4.45 eV might be affected by the redistribution of photoinduced electron and hole populations. The global analysis of the experimental data produces a fitted TA map in Figure 1g that accurately reproduces the measured results (see the residuals in Figure S9), and the resulting DASs are presented in Figure 1h. Interestingly, the DAS associated with the sub-picosecond lifetime exhibits a derivative-like spectral response, similar to DAS1 in the visible spectral region (Figure 1d). This suggests the occurrence of a band edge renormalization effect of the higher-energy band at the M symmetry point. The DAS2 and DAS3 display similar profiles, but it is important to note the different amplitude ratio of the ∼3.8 and ∼3.4 eV components, as well as the red-shifted positive signal observed in DAS3, which resembles the spectral traces at early and late delay times, respectively. Taken together, these findings indicate that the decay of the transients involves multiple kinetic components that share similar spectral signatures.

### 2.4 Temperature-Dependent Spectra

**Figure 2:**
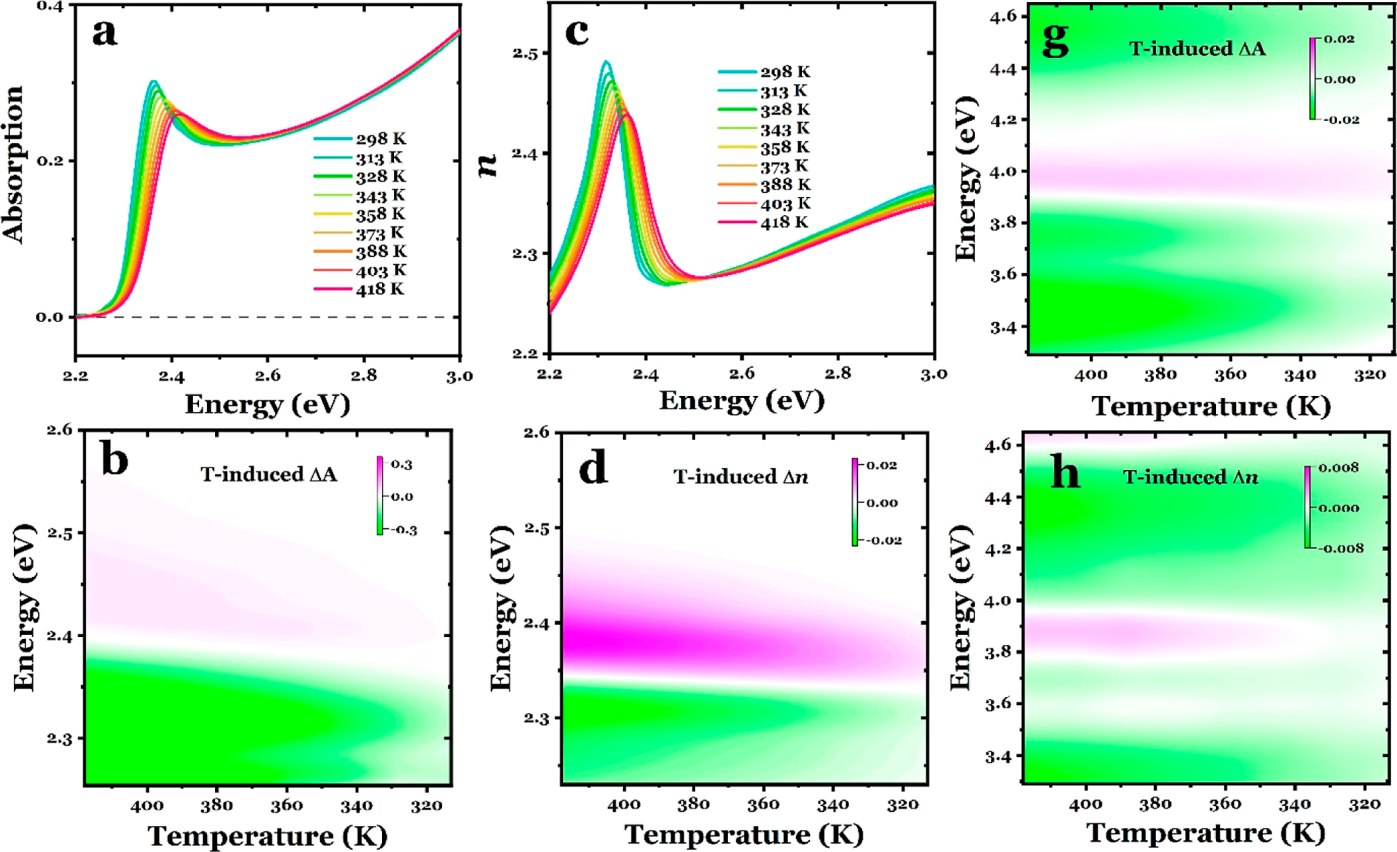
Thermal-induced responses in the MAPbBr_3_ perovskite in the visible and mid-to-deep-UV spectral regions. (a) Temperature-dependent absorption in the visible spectral region extracted from VASE. (b) Differential absorption (Δ*A*) of MAPbBr_3_ in the visible spectral region, obtained by subtracting the absorption measured at high temperatures from that at room temperature (298 K). (c) Temperature-dependent refractive index in the visible spectral region. (d) Differential refractive index (Δ*n*) in the visible spectral region, obtained by subtracting the refractive indexes measured at high temperatures from that at room temperature (298 K). (g) Differential absorption (Δ*A*) of MAPbBr_3_ in the mid-to-deep-UV spectral region. (h) Differential refractive index (Δ*n*) in the mid-to-deep-UV spectral region.

To investigate the thermal characteristics of the MAPbBr_3_ perovskite, a single crystal was used as a benchmark material and subject to heating using in situ VASE at temperatures ranging from 298 to 418 K in intervals of 15 K (Figure S10). The extracted absorption coefficients and refractive indexes at each measured temperature are shown in Figure 2a,c. Both quantities exhibit a continuous decrease in intensity and a spectral blue shift, which aligns well with DFT calculations of temperature (T)-dependent BGs^27,56^ (Figure S11), where the lattice temperatures are simulated by varying the lattice parameters in increments of 0.5, 1.0, 1.5, and 2.0%.

The spectral changes are better highlighted by subtracting the absorption and refractive index values measured at high temperatures from those at room temperature (298 K). The resulting differential changes in the absorption (Δ*A*) and refractive index (Δ*n*) are plotted as a function of energy and temperature in Figure 2b,d for the visible region and in Figure 2g,h for the UV spectral region. Remarkably, the T-induced differential absorption (TDA) shows a response similar to the photoexcited case in both low- and high-energy bands (Figure S12), while the T-induced Δ*n* exhibits a red-shifted spectral feature (note the position of zero-crossing points) in both regions, with an amplitude more than 1 order of magnitude smaller than that of Δ*A*. These observations emphasize the correlation between the behaviors under photoexcitation and upon heating.

## 3 Discussion

**Figure 3:**
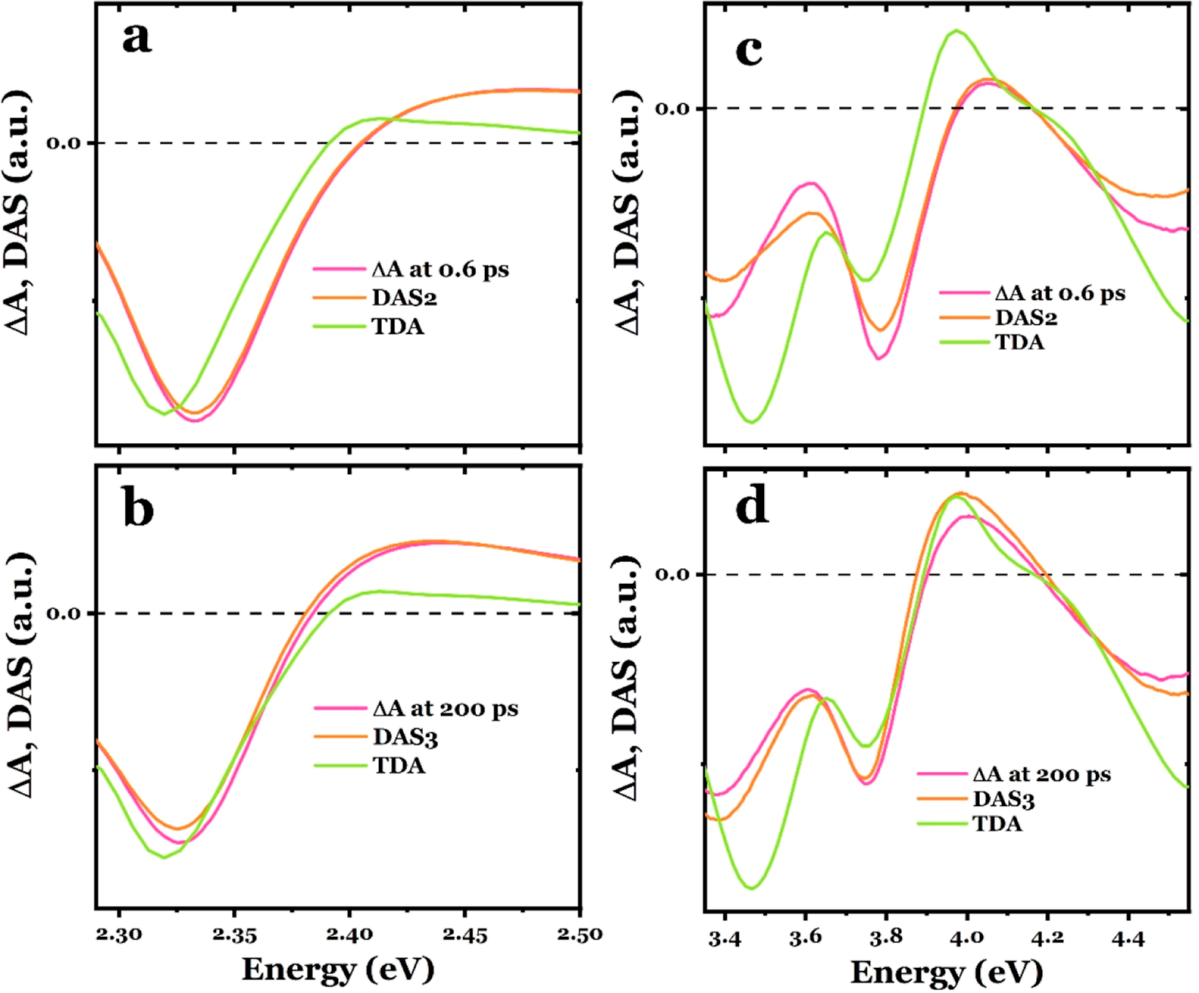
Comparison of photo- and thermal-induced spectral responses in the MAPbBr_3_ perovskite. (a) Visible spectral region. The comparison comprises the transient spectral trace at 0.6 ps, the DAS2 spectrum, and the TDA spectrum at 373 K. (b) Visible spectral region, comprising the transient spectral trace at 200 ps, the DAS3 spectrum, and the TDA spectrum at 373 K. (c) Mid-to-deep-UV spectral region, including the transient spectral trace at 0.6 ps, the DAS2 spectrum, and the TDA spectrum at 373 K. (d) Mid-to-deep-UV spectral region, including the transient spectral trace at 200 ps, the DAS3 spectrum, and the TDA spectrum at 373 K.

Based on the above, we compare the TA spectral traces at early and late time scales (e.g., 0.6 and 200 ps), the DAS2 and DAS3 spectra, and the TDA at 373 K in Figure 3a,b for the visible spectral region and in Figure 3c,d for the UV spectral region. The TDA spectrum at 373 K was selected due to its close resemblance to the transient spectra and an estimate of the lattice temperature increase in photoexcited experiments (Note S3). All spectra were rescaled for clarity. From Figure 3a,c, the DAS2 spectrum reflects the electronic component of the TA signal well, and both the TA spectrum at 0.6 ps and DAS2 differ from the TDA spectrum in terms of band positions in both spectral regions. In Figure 3b,d, the TDA spectra reflect better the overall spectral features of the photoinduced spectra at 200 ps time delay, in terms of the sign of the bands, their respective positions, and ratios, although some deviations are also visible. The DAS3 spectrum, which corresponds to the largest lifetime TA signal amplitude, also exhibits a similar profile to the 200 ps TA trace and the TDA spectrum at 373 K. These observations show that at long time delays after photoexcitation, the TA spectral traces share significant similarities with thermal responses. However, the T-induced changes in the refractive index show a complete misalignment with the spectral signatures observed in the TAs (Figure S13), indicating that the thermal-induced responses in the transmitted transient signal of MAPbBr_3_ are primarily manifested as changes in the absorption coefficient rather than the refractive index.

In order to reconstruct the photoinduced TA spectra by utilizing the signal amplitudes at different delay stages, we introduce two evolution parameters, *m* and *n*, which reflect the spectral weights of the electronic and thermal contributions, respectively. At early times following photoexcitation, thermal effects are negligible, and the transient behavior can be reproduced by combining DAS1 and *m* × DAS2 (shown on the left sides of Figure S14a,b). We varied the value of *m* from 1 to 5 to simulate the relative increasing contribution from DAS2 over time. As we move to the later stages, typically after 1 ps, the BGR, represented by DAS1, diminishes, allowing the simulation of the complex spectral evolution in the long-term component using 5 × DAS2 + *n* × DAS3. Here, we explored values of *n* ranging from 1 to 15 (shown on the right sides of Figure S14a,b). Remarkably, in both the visible and UV spectral regions, the reconstructed differential absorption map reproduced the experimental TA profiles well as their temporal evolution. We chose *m* = 1.7 and *n* = 1 and 10 to represent the early, intermediate, and long-term delay times, respectively. The reconstructed absorption changes in Figure S15 (visible region) and S16 (UV region) exhibit great similarities with the TA spectral traces recorded at 100 fs, 1 ps, and 100 ps. Notably, the positive features at ∼2.2 eV and the negative peak variations at ∼3.4 and 3.8 eV were also accurately reproduced. This enabled us to perform spectral response fitting at each delay time, using different combinations of DASs. Since we are interested in the contribution of thermal vs electronic effects, we fitted the data from 1 ps onward, thus excluding the BGR signal. The principal shape of the electronic signal is preserved by DAS2, and the fitting procedure involved using *a* × DAS2 + *b* × DAS3. Figures S17 and S18 depict the experimental and fitted spectral traces in the visible and UV probe regions, respectively, showcasing the remarkable consistency and reproducibility achieved through the GLA and spectral trace fitting. This robust fitting approach serves as a basis for further analysis, allowing for the replacement of DAS3 by the TDA spectrum.

**Figure 4:**
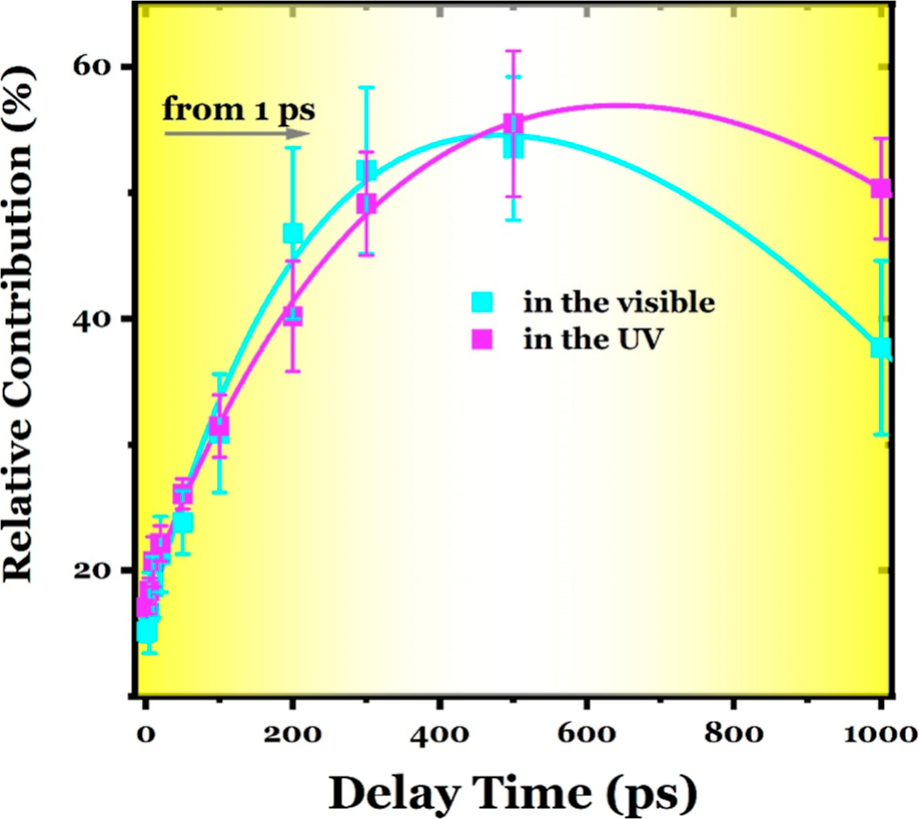
Relative thermal contributions in the TA signal. The contributions of electronic and thermal effects in the TA signal were quantified from 1 ps onward, where the BGR signals have completely vanished. At 1 ps, the thermal contribution to the overall transient signal is approximately 15% in both the visible and UV probe regions. These contributions initially increase to ∼55% at 500 ps but subsequently decrease to ∼35–50% at 1000 ps. The error bars were determined based on the upper and lower limits of the fit values.

The revised fits using various ratios of combinations of DAS2 and TDA (*a* × DAS2 + *b* × TDA) are shown in Figures S19 and S20. We assume that the electronic and thermal contributions to the transient amplitude are independent, the thermal contributions to the TA signal are characterized by the TDA spectrum, and its relative contribution is determined by calculating *b*/(*a* + *b*) × 100%. In the fit of the visible-probed spectral traces, the energy range was truncated at 2.25 eV to ensure consistency with the TDA spectrum (as explained in Note S4). The thermal contributions to the TA signal are illustrated in Figure 4 for the low-energy BG transition in the visible region and the high-energy interband transitions in the UV region. At a 1 ps time delay, the thermal contribution to the overall TA signal is approximately 15% (correspondingly, the relative electronic contribution is ∼85%) in both the visible and UV regions, gradually increasing to ∼55% at ∼500 ps. Subsequently, it exhibits a decrease of ∼35–50% at 1 ns. This temporal evolution, marked by an increase followed by a decrease of the thermal contribution, can be qualitatively modeled using two exponential functions with time constants of 250 ± 40 ps (τ_1_) and 990 ± 200 ps (τ_2_) for the visible region and 390 ± 20 ps (τ_1_) and 925 ± 185 ps (τ_2_) for the UV region. The small discrepancy between the low- and high-energy bands could be ascribed to different mechanisms involved in generating the TA signal between the BG transition and higher-energy interband transitions, such as Pauli blocking at the BG (R symmetry point) and Coulomb screening in the high-energy bands (M–R and Γ–X symmetry points). In the low-energy band, despite the well-documented excitonic bleach induced by band filling,^57,58^Figure S21 demonstrates the broadening of the width of the excitonic line shape with increasing pump fluence, indicating the influence of screening by photogenerated electrons at the BG transition. In Figure S22, the differential absorption was reconstructed using a method similar to that in Figure S14, but focusing on the mid-to-long-term behavior and varying the value of *n* from 1 to 6 and 6 to 4. The corresponding relative electronic contributions for different evolution numbers are also shown in Figure S22b, providing a good simulation of the experimental absorption difference changes with delay time. In summary, the probing in the visible and UV spectral regions unambiguously reveals that photoexcited charge carriers transfer their energy to the lattice within the 200–400 ps time scale, followed by heat dissipation that is seen in a decreasing relative thermal contribution on the time scale of hundreds of picoseconds to nanoseconds.

From the above, it is evident that the spectral response of photoexcited perovskite materials exhibits a substantial thermal contribution. The energy exchange between photoexcited charge carriers and the lattice within a sub-nanosecond time window demonstrates a temporal evolution of the spectral weight of the thermal component, characterized by an initial increase followed by a subsequent decrease. Initially, there is a continuous transfer of energy from electrons and holes to the lattice, resulting in a progressive enhancement of the thermal contribution to the overall TA signal over a time frame of 200–400 ps in both probed spectral regions. Subsequently, as the thermal contribution fades away, the TA signal is dominated by electronic contributions. This is due to the fact that electronic relaxation, which mostly takes place via radiative electron–hole recombination, occurs at much slower rates of tens to hundreds of ns.^59^ However, it is important to note that while the relative electronic/thermal contribution to the overall TA signal increases/decreases at longer delay times, both the electronic and thermal effects are in fact diminishing as a function of the delay time.

## 4 Conclusions

In this study, we conducted broadband TA spectroscopy spanning the visible to mid-to-deep-UV ranges on MAPbBr_3_ thin films as well as the temperature-dependent VASE and DFT calculations. The use of deep-UV detection enables the exploration of high-energy charge excitations around various symmetry points within the BZ; thus, it opens a novel spectral window and confers a significant advantage compared to conventional visible to terahertz probes. Our investigation reveals that the photoinduced spectral features possess a significant thermal origin and cannot be attributed solely to electronic effects. Furthermore, we disentangle the relative weights of the thermal and electronic contributions and their evolutions at different delay times using combinations of DAS and TDA spectra. The results demonstrate that as electrons and holes transfer their energy to the lattice, the thermal contribution to the overall TA signal exhibit an initial increase followed by a subsequent decrease behavior of spectral weight as a function of time delay, i.e., initially increases from ∼15% at 1 ps to ∼55% at 500 ps but subsequently decreases to ∼35–50% at 1 ns.

Gaining insights into such ultrafast energy transfer behavior holds great potential for enhancing the photoelectronic conversion efficiency in perovskite-based photovoltaic and optoelectronic devices across diverse application scenarios. This is particularly important for determining optimal parameters based on the effective operational duration of photogenerated electrons and holes, with the aim of maximizing the utilization of charge carriers before their complete energy dissipation into the lattice. Furthermore, the results emphasize the necessity for caution when attributing the spectral responses solely to the behavior of photogenerated electrons and holes. Moreover, it is also imperative to consider additional factors that can influence the energy transfer process, such as the type of the perovskite material, sample morphology, pump photon energy, and intensity. This calls for further detailed experimental and theoretical investigations to establish a standardized energy transfer rate profile under various conditions for different perovskite materials. Such endeavors will unravel the reasons behind the less than optimal utilization efficiency of photogenerated charge carriers in perovskite solar cell materials.

## Supporting Information Available

Experimental procedures; characterization data; additional analysis; schematic diagram of photon-induced and thermally excited differential absorption; absorption spectrum of a MAPbBr_3_ single crystal; calculated energy band diagram; normalized TA spectral traces at 0.2, 1, 10, 100, and 1000 ps; time traces probed at 2.35 and 4.45 eV; fitting residuals of the time–energy map; time traces of the rising TA signals; VASE data of the MAPbBr_3_ perovskite; calculated BGs of the MAPbBr_3_ perovskite; comparison of the photon-induced and thermally induced Δ*A* profiles; comparison of photon-induced transients at 0.6 and 200 ps and thermally induced Δ*n* at 373 K; reconstructed differential absorption map using various ratios of combinations of m and n; experimentally visible- and UV-probed spectral traces and their fittings; reconstructed differential absorption map using various ratios of combinations of DAS2 and TDA; and schematic representation illustrating the thermal decay of laser-induced hot spot within MAPbBr_3_ films (PDF)

## References

[ref1] Zewail A. H. Femtochemistry: Atomic-Scale Dynamics of the Chemical Bond. J. Phys. Chem. A 2000, 104 (24), 5660–5694. 10.1021/jp001460h. 10934390

[ref2] Chergui M. Ultrafast Photophysics of Transition Metal Complexes. Acc. Chem. Res. 2015, 48 (3), 801–808. 10.1021/ar500358q. 25646968

[ref3] Kambhampati P. Learning about the Structural Dynamics of Semiconductor Perovskites from Electron Solvation Dynamics. J. Phys. Chem. C 2021, 125 (43), 23571–23586. 10.1021/acs.jpcc.1c07445.

[ref4] Wang R.; Huang T.; Xue J.; Tong J.; Zhu K.; Yang Y. Prospects for Metal Halide Perovskite-Based Tandem Solar Cells. Nat. Photonics 2021, 15 (6), 411–425. 10.1038/s41566-021-00809-8.

[ref5] Thouin F.; Valverde-Chávez D. A.; Quarti C.; Cortecchia D.; Bargigia I.; Beljonne D.; Petrozza A.; Silva C.; Srimath Kandada A. R. Phonon Coherences Reveal the Polaronic Character of Excitons in Two-Dimensional Lead Halide Perovskites. Nat. Mater. 2019, 18 (4), 349–356. 10.1038/s41563-018-0262-7. 30643234

[ref6] Yang J.; Wen X.; Xia H.; Sheng R.; Ma Q.; Kim J.; Tapping P.; Harada T.; Kee T. W.; Huang F.; et al. Acoustic-Optical Phonon up-Conversion and Hot-Phonon Bottleneck in Lead-Halide Perovskites. Nat. Commun. 2017, 8 (1), 14120 10.1038/ncomms14120. 28106061 PMC5263885

[ref7] Wang L.; McCleese C.; Kovalsky A.; Zhao Y.; Burda C. Femtosecond Time-Resolved Transient Absorption Spectroscopy of CH_3_NH_3_PbI_3_ Perovskite Films: Evidence for Passivation Effect of PbI_2_. J. Am. Chem. Soc. 2014, 136 (35), 12205–12208. 10.1021/ja504632z. 25145978

[ref8] Guo J.; Ohkita H.; Benten H.; Ito S. Near-IR Femtosecond Transient Absorption Spectroscopy of Ultrafast Polaron and Triplet Exciton Formation in Polythiophene Films with Different Regioregularities. J. Am. Chem. Soc. 2009, 131 (46), 16869–16880. 10.1021/ja906621a. 19886624

[ref9] Oppermann M.; Zinna F.; Lacour J.; Chergui M. Chiral Control of Spin-Crossover Dynamics in Fe (II) Complexes. Nat. Chem. 2022, 14 (7), 739–745. 10.1038/s41557-022-00933-0. 35618767

[ref10] Oppermann M.; Bauer B.; Rossi T.; Zinna F.; Helbing J.; Lacour J.; Chergui M. Ultrafast Broadband Circular Dichroism in the Deep Ultraviolet. Optica 2019, 6 (1), 56–60. 10.1364/OPTICA.6.000056.

[ref11] Auböck G.; Consani C.; Monni R.; Cannizzo A.; van Mourik F.; Chergui M. Femtosecond Pump/Supercontinuum-Probe Setup with 20 kHz Repetition Rate. Rev. Sci. Instrum. 2012, 83 (9), 093105 10.1063/1.4750978. 23020360

[ref12] Baldini E.; Palmieri T.; Rossi T.; Oppermann M.; Pomarico E.; Auböck G.; Chergui M. Interfacial Electron Injection Probed by a Substrate-Specific Excitonic Signature. J. Am. Chem. Soc. 2017, 139 (33), 11584–11589. 10.1021/jacs.7b06322. 28762734

[ref13] Smolin S. Y.; Choquette A. K.; Wang J.; May S. J.; Baxter J. B. Distinguishing Thermal and Electronic Effects in Ultrafast Optical Spectroscopy Using Oxide Heterostructures. J. Phys. Chem. C 2018, 122 (1), 115–123. 10.1021/acs.jpcc.7b09592.

[ref14] Hayes D.; Hadt R. G.; Emery J. D.; Cordones A. A.; Martinson A. B.; Shelby M. L.; Fransted K. A.; Dahlberg P. D.; Hong J.; Zhang X.; et al. Electronic and Nuclear Contributions to Time-Resolved Optical and X-Ray Absorption Spectra of Hematite and Insights into Photoelectrochemical Performance. Energy Environ. Sci. 2016, 9 (12), 3754–3769. 10.1039/c6ee02266a.

[ref15] Bennett B. R.; Soref R. A.; Del Alamo J. A. Carrier-Induced Change in Refractive Index of InP, GaAs and InGaAsP. IEEE J. Quantum Electron. 1990, 26 (1), 113–122. 10.1109/3.44924.

[ref16] Sheu Y.-M.; Trugman S. A.; Park Y.-S.; Lee S.; Yi H. T.; Cheong S.-W.; Jia Q. X.; Taylor A. J.; Prasankumar R. P. Ultrafast Carrier Dynamics and Radiative Recombination in Multiferroic BiFeO_3_. Appl. Phys. Lett. 2012, 100 (24), 242904 10.1063/1.4729423.

[ref17] Sabbah A. J.; Riffe D. M. Femtosecond Pump-Probe Reflectivity Study of Silicon Carrier Dynamics. Phys. Rev. B 2002, 66 (16), 165217 10.1103/PhysRevB.66.165217.

[ref18] Xing G.; Mathews N.; Sun S.; Lim S. S.; Lam Y. M.; Grätzel M.; Mhaisalkar S.; Sum T. C. Long-Range Balanced Electron-and Hole-Transport Lengths in Organic-Inorganic CH_3_NH_3_PbI_3_. Science 2013, 342 (6156), 344–347. 10.1126/science.1243167. 24136965

[ref19] Dai L.; Deng Z.; Auras F.; Goodwin H.; Zhang Z.; Walmsley J. C.; Bristowe P. D.; Deschler F.; Greenham N. C. Slow Carrier Relaxation in Tin-Based Perovskite Nanocrystals. Nat. Photonics 2021, 15 (9), 696–702. 10.1038/s41566-021-00847-2.

[ref20] Plankl M.; Faria Junior P. E.; Mooshammer F.; Siday T.; Zizlsperger M.; Sandner F.; Schiegl F.; Maier S.; Huber M. A.; Gmitra M.; Fabian J.; Boland J. L.; Cocker T. L.; Huber R. Subcycle Contact-Free Nanoscopy of Ultrafast Interlayer Transport in Atomically Thin Heterostructures. Nat. Photonics 2021, 15 (8), 594–600. 10.1038/s41566-021-00813-y.

[ref21] Brauer J. C.; Lee Y. H.; Nazeeruddin M. K.; Banerji N. Charge Transfer Dynamics from Organometal Halide Perovskite to Polymeric Hole Transport Materials in Hybrid Solar Cells. J. Phys. Chem. Lett. 2015, 6 (18), 3675–3681. 10.1021/acs.jpclett.5b01698. 26722741

[ref22] Senanayak S. P.; Dey K.; Shivanna R.; Li W.; Ghosh D.; Zhang Y.; Roose B.; Zelewski S. J.; Andaji-Garmaroudi Z.; Wood W.; Tiwale N.; MacManus-Driscoll J. L.; Friend R. H.; Stranks S. D.; Sirringhaus H. Charge Transport in Mixed Metal Halide Perovskite Semiconductors. Nat. Mater. 2023, 22 (2), 216–224. 10.1038/s41563-022-01448-2. 36702888

[ref23] Li W.; Zheng J.; Hu B.; Fu H.-C.; Hu M.; Veyssal A.; Zhao Y.; He J.-H.; Liu T. L.; Ho-Baillie A.; Jin S. High-Performance Solar Flow Battery Powered by a Perovskite/Silicon Tandem Solar Cell. Nat. Mater. 2020, 19 (12), 1326–1331. 10.1038/s41563-020-0720-x. 32661381

[ref24] Wang W.; Ghosh T.; Yan H.; Erofeev I.; Zhang K.; Loh K. P.; Mirsaidov U. The Growth Dynamics of Organic-Inorganic Metal Halide Perovskite Films. J. Am. Chem. Soc. 2022, 144 (39), 17848–17856. 10.1021/jacs.2c06022. 36130403

[ref25] Li Y.; Lai R.; Luo X.; Liu X.; Ding T.; Lu X.; Wu K. On the Absence of a Phonon Bottleneck in Strongly Confined CsPbBr_3_ Perovskite Nanocrystals. Chem. Sci. 2019, 10 (23), 5983–5989. 10.1039/C9SC01339C. 31360405 PMC6566378

[ref26] Wright A. D.; Verdi C.; Milot R. L.; Eperon G. E.; Pérez-Osorio M. A.; Snaith H. J.; Giustino F.; Johnston M. B.; Herz L. M. Electron-Phonon Coupling in Hybrid Lead Halide Perovskites. Nat. Commun. 2016, 7 (1), 11755 10.1038/ncomms11755. PMC4894981 27225329

[ref27] Mannino G.; Deretzis I.; Smecca E.; La Magna A.; Alberti A.; Ceratti D.; Cahen D. Temperature-Dependent Optical Band Gap in CsPbBr_3_, MAPbBr_3_, and FAPbBr_3_ Single Crystals. J. Phys. Chem. Lett. 2020, 11 (7), 2490–2496. 10.1021/acs.jpclett.0c00295. 32148047 PMC7467746

[ref28] Park S.; Seo Y.-S.; Ahn C. W.; Woo W. S.; Kyhm J.; Lee S. A.; Kim I. W.; Hwang J. Temperature-Dependent Optical Properties of Hybrid Organic-Inorganic Perovskite Single Crystals (CH_3_NH_3_PbI_3_ and CH_3_NH_3_PbBr_3_). J. Phys. D: Appl. Phys. 2019, 52 (33), 335302 10.1088/1361-6463/ab20fa.

[ref29] Strandell D. P.; Kambhampati P. Light Emission from CsPbBr_3_ Metal Halide Perovskite Nanocrystals Arises from Dual Emitting States with Distinct Lattice Couplings. Nano Lett. 2023, 23 (23), 11330–11336. 10.1021/acs.nanolett.3c03975. 38088142

[ref30] Ghosh A.; Strandell D. P.; Kambhampati P. A Spectroscopic Overview of the Differences between the Absorbing States and the Emitting States in Semiconductor Perovskite Nanocrystals. Nanoscale 2023, 15 (6), 2470–2487. 10.1039/D2NR05698D. 36691921

[ref31] Wang L.; Rossi T.; Oppermann M.; Bauer B.; Mewes L.; Zare D.; Chow T. H.; Wang J.; Chergui M. Slow Charge Carrier Relaxation in Gold Nanoparticles. J. Phys. Chem. C 2020, 124 (44), 24322–24330. 10.1021/acs.jpcc.0c07755.

[ref32] Wang L.; Oppermann M.; Puppin M.; Bauer B.; Chow T. H.; Wang J.; Chergui M. Interband Transition Probing of Coherent Acoustic Phonons of Gold/Metal Oxide Core-Shell Nanoparticles. Appl. Phys. Lett. 2023, 122 (8), 082201 10.1063/5.0139466.

[ref33] Wang L.; Chow T. H.; Oppermann M.; Wang J.; Chergui M. Giant Two-Photon Absorption of Anatase TiO_2_ in Au/TiO_2_ Core-Shell Nanoparticles. Photonics Res. 2023, 11 (7), 1303–1313. 10.1364/PRJ.487784.

[ref34] Peter Y. U.; Cardona M. Fundamentals of Semiconductors: Physics and Materials Properties; Springer Science & Business Media, 2010.

[ref35] Slavov C.; Hartmann H.; Wachtveitl J. Implementation and Evaluation of Data Analysis Strategies for Time-Resolved Optical Spectroscopy. Anal. Chem. 2015, 87 (4), 2328–2336. 10.1021/ac504348h. 25590674

[ref36] Leguy A. M. A.; Azarhoosh P.; Alonso M. I.; Campoy-Quiles M.; Weber O. J.; Yao J.; Bryant D.; Weller M. T.; Nelson J.; Walsh A.; van Schilfgaarde M.; Barnes P. R. F. Experimental and Theoretical Optical Properties of Methylammonium Lead Halide Perovskites. Nanoscale 2016, 8 (12), 6317–6327. 10.1039/C5NR05435D. 26477295

[ref37] Mosconi E.; Umari P.; De Angelis F. Electronic and Optical Properties of MAPbX_3_ Perovskites (X = I, Br, Cl): A Unified DFT and GW Theoretical Analysis. Phys. Chem. Chem. Phys. 2016, 18 (39), 27158–27164. 10.1039/c6cp03969c. 27711483

[ref38] Niedzwiedzki D. M.; Kouhnavard M.; Diao Y.; D’Arcy J. M.; Biswas P. Spectroscopic Investigations of Electron and Hole Dynamics in MAPbBr_3_ Perovskite Film and Carrier Extraction to PEDOT Hole Transport Layer. Phys. Chem. Chem. Phys. 2021, 23 (23), 13011–13022. 10.1039/d1cp00658d. 34095927

[ref39] Elmelund T.; Scheidt R. A.; Seger B.; Kamat P. V. Bidirectional Halide Ion Exchange in Paired Lead Halide Perovskite Films with Thermal Activation. ACS Energy Lett. 2019, 4 (8), 1961–1969. 10.1021/acsenergylett.9b01280.

[ref40] Li M.; Wei Q.; Muduli S. K.; Yantara N.; Xu Q.; Mathews N.; Mhaisalkar S. G.; Xing G.; Sum T. C. Enhanced Exciton and Photon Confinement in Ruddlesden-Popper Perovskite Microplatelets for Highly Stable Low-Threshold Polarized Lasing. Adv. Mater. 2018, 30 (23), 1707235 10.1002/adma.201707235. 29709082

[ref41] Grancini G.; Srimath Kandada A. R.; Frost J. M.; Barker A. J.; De Bastiani M.; Gandini M.; Marras S.; Lanzani G.; Walsh A.; Petrozza A. Role of Microstructure in the Electron-Hole Interaction of Hybrid Lead Halide Perovskites. Nat. Photonics 2015, 9 (10), 695–701. 10.1038/nphoton.2015.151. 26442125 PMC4591469

[ref42] Li M.; Bhaumik S.; Goh T. W.; Kumar M. S.; Yantara N.; Grätzel M.; Mhaisalkar S.; Mathews N.; Sum T. C. Slow Cooling and Highly Efficient Extraction of Hot Carriers in Colloidal Perovskite Nanocrystals. Nat. Commun. 2017, 8 (1), 14350 10.1038/ncomms14350. 28176882 PMC5309769

[ref43] Palmieri T.; Baldini E.; Steinhoff A.; Akrap A.; Kollár M.; Horváth E.; Forró L.; Jahnke F.; Chergui M. Mahan Excitons in Room-Temperature Methylammonium Lead Bromide Perovskites. Nat. Commun. 2020, 11 (1), 850 10.1038/s41467-020-14683-5. 32051405 PMC7016123

[ref44] Miyata K.; Meggiolaro D.; Trinh M. T.; Joshi P. P.; Mosconi E.; Jones S. C.; De Angelis F.; Zhu X.-Y. Large Polarons in Lead Halide Perovskites. Sci. Adv. 2017, 3 (8), e1701217 10.1126/sciadv.1701217. 28819647 PMC5553817

[ref45] Liu J.; Leng J.; Wang S.; Zhang J.; Jin S. Artifacts in Transient Absorption Measurements of Perovskite Films Induced by Transient Reflection from Morphological Microstructures. J. Phys. Chem. Lett. 2019, 10 (1), 97–101. 10.1021/acs.jpclett.8b03704. 30985141

[ref46] Wu B.; Nguyen H. T.; Ku Z.; Han G.; Giovanni D.; Mathews N.; Fan H. J.; Sum T. C. Discerning the Surface and Bulk Recombination Kinetics of Organic-Inorganic Halide Perovskite Single Crystals. Adv. Energy Mater. 2016, 6 (14), 1600551 10.1002/aenm.201600551.

[ref47] Diroll B. T. Temperature-Dependent Intraband Relaxation of Hybrid Perovskites. J. Phys. Chem. Lett. 2019, 10 (18), 5623–5628. 10.1021/acs.jpclett.9b02320. 31502463

[ref48] Qin J.; Tang Y.; Zhang J.; Shen T.; Karlsson M.; Zhang T.; Cai W.; Shi L.; Ni W.-X.; Gao F. From Optical Pumping to Electrical Pumping: The Threshold Overestimation in Metal Halide Perovskites. Mater. Horiz. 2023, 10 (4), 1446–1453. 10.1039/D2MH01382G. 36789680

[ref49] Fu J.; Xu Q.; Han G.; Wu B.; Huan C. H. A.; Leek M. L.; Sum T. C. Hot Carrier Cooling Mechanisms in Halide Perovskites. Nat. Commun. 2017, 8 (1), 1300 10.1038/s41467-017-01360-3. 29101381 PMC5670184

[ref50] Baker H.; Perez C. M.; Sonnichsen C.; Strandell D.; Prezhdo O. V.; Kambhampati P. Breaking Phonon Bottlenecks through Efficient Auger Processes in Perovskite Nanocrystals. ACS Nano 2023, 17 (4), 3913–3920. 10.1021/acsnano.2c12220. 36796027

[ref51] Brosseau P.; Ghosh A.; Seiler H.; Strandell D.; Kambhampati P. Exciton-Polaron Interactions in Metal Halide Perovskite Nanocrystals Revealed via Two-Dimensional Electronic Spectroscopy. J. Chem. Phys. 2023, 159 (18), 184711 10.1063/5.0173369. 37962451

[ref52] van Stokkum I. H. M.; Larsen D. S.; van Grondelle R. Global and Target Analysis of Time-Resolved Spectra. Biochim. Biophys. Acta, Bioenerg. 2004, 1657 (2–3), 82–104. 10.1016/j.bbabio.2004.04.011. 15238266

[ref53] Price M. B.; Butkus J.; Jellicoe T. C.; Sadhanala A.; Briane A.; Halpert J. E.; Broch K.; Hodgkiss J. M.; Friend R. H.; Deschler F. Hot-Carrier Cooling and Photoinduced Refractive Index Changes in Organic-Inorganic Lead Halide Perovskites. Nat. Commun. 2015, 6 (1), 8420 10.1038/ncomms9420. 26404048 PMC4598728

[ref54] Sung J.; Schnedermann C.; Ni L.; Sadhanala A.; Chen R.; Cho C.; Priest L.; Lim J. M.; Kim H.-K.; Monserrat B.; et al. Long-Range Ballistic Propagation of Carriers in Methylammonium Lead Iodide Perovskite Thin Films. Nat. Phys. 2020, 16 (2), 171–176. 10.1038/s41567-019-0730-2.

[ref55] Rossi D.; Wang H.; Dong Y.; Qiao T.; Qian X.; Son D. H. Light-Induced Activation of Forbidden Exciton Transition in Strongly Confined Perovskite Quantum Dots. ACS Nano 2018, 12 (12), 12436–12443. 10.1021/acsnano.8b06649. 30521756

[ref56] Saxena R.; Kangsabanik J.; Kumar A.; Shahee A.; Singh S.; Jain N.; Ghorui S.; Kumar V.; Mahajan A. V.; Alam A.; Kabra D. Contrasting Temperature Dependence of the Band Gap in CH_3_NH_3_PbX_3_ (X = I, Br, Cl): Insight from Lattice Dilation and Electron-Phonon Coupling. Phys. Rev. B 2020, 102 (8), 081201 10.1103/PhysRevB.102.081201.

[ref57] Guzelturk B.; Winkler T.; Van de Goor T. W. J.; Smith M. D.; Bourelle S. A.; Feldmann S.; Trigo M.; Teitelbaum S. W.; Steinrück H. G.; de la Pena G. A.; Alonso-Mori R.; Zhu D.; Sato T.; Karunadasa H. I.; Toney M. F.; Deschler F.; Lindenberg A. M. Visualization of Dynamic Polaronic Strain Fields in Hybrid Lead Halide Perovskites. Nat. Mater. 2021, 20 (5), 618–623. 10.1038/s41563-020-00865-5. 33398119

[ref58] Yang Y.; Ostrowski D. P.; France R. M.; Zhu K.; van de Lagemaat J.; Luther J. M.; Beard M. C. Observation of a Hot-Phonon Bottleneck in Lead-Iodide Perovskites. Nat. Photonics 2016, 10 (1), 53–59. 10.1038/nphoton.2015.213.

[ref59] Lee K. J.; Wei R.; Wang Y.; Zhang J.; Kong W.; Chamoli S. K.; Huang T.; Yu W.; ElKabbash M.; Guo C. Gigantic Suppression of Recombination Rate in 3D Lead-Halide Perovskites for Enhanced Photodetector Performance. Nat. Photonics 2023, 17 (3), 236–243. 10.1038/s41566-022-01151-3.

