# Supplementary material for: Disentangling Thermal from Electronic Contributions in the Spectral Response of Photoexcited Perovskite Materials
Source: J Am Chem Soc. 2024 Feb 15;146(8):5393–401. doi: 10.1021/jacs.3c12832 (PMC10910496; doi:10.1021/jacs.3c12832)
Supplement: Supplementary file 1 — ja3c12832_si_001.pdf [file ja3c12832_si_001.pdf]

Supplementary Information

Disentangling Thermal from Electronic Contributions in the Spectral Response of Photo-excited Perovskite Materials

Lijie Wang[1,2], Razan Nughays[2], Thomas C. Rossi[1], Malte Oppermann[1], Wojciech Ogieglo[2], Tieyuan Bian[3], Chun-Hua Shih[4], Tzung-Fang Guo[4], Ingo Pinnau[2], Jun Yin[3], Osman M. Bakr[5], Omar F. Mohammed[2,5*] & Majed Chergui[1*]

[1]Lausanne Centre for Ultrafast Science (LACUS), ISIC, École Polytechnique Fédérale de Lausanne (EPFL), CH-1015 Lausanne, Switzerland.

[2]Advanced Membranes and Porous Materials Center (AMPM), Division of Physical Science and Engineering, King Abdullah University of Science and Technology, Thuwal 23955-6900, Kingdom of Saudi Arabia.

[3]Department of Applied Physics, The Hong Kong Polytechnic University, Kowloon 999077 Hong Kong, P. R. China.

[4]Department of Photonics, National Cheng Kung University, Tainan 701, Taiwan ROC.

[5]KAUST Catalysis Center, Division of Physical Sciences and Engineering, King Abdullah University of Science and Technology, Thuwal 23955-6900, Kingdom of Saudi Arabia.

Correspondance Email: *omar.abdelsaboor@kaust.edu.sa*; *majed.chergui@epfl.ch*.

Experimental Methods

Sample preparation and characterization. For $MAPbBr_3$ films used for TA experiments, prior to the deposition, the quartz substrates underwent a sequential cleaning process. Firstly, the substrates were subjected to ultrasonic treatment in detergent, de-ionized water, acetone, and isopropyl alcohol. After drying, the cleaned substrates were further treated with UV-ozone (Model: 42, Jelight, USA) for 25 minutes. The $MAPbBr_3$ precursor solution was prepared by combining 0.015 g of MABr (Dyesol) with 0.0481 g of $PbBr_2$ (Sigma-Aldrich, 99.999%) in an anhydrous dimethyl sulfoxide (DMSO, Sigma-Aldrich) solution (1070 µl) at 60°C. The solution was stirred for 12 hours. To fabricate the $MAPbBr_3$ perovskite thin film, a consecutive two-step spin-coating process was employed. The solution was spin-coated onto the quartz substrates at 500 rpm for 7 seconds, followed by a spin-coating at 4000 rpm for 70 seconds. Additionally, at 43 seconds during the spin coating, 250 µl of chloroform solvent was dropped onto the surface of the precursor film. Subsequently, the $MAPbBr_3$ perovskite film was annealed on a hotplate at 70°C for 10 minutes. It is noteworthy that all the procedures for preparing the $MAPbBr_3$ precursor solution and films were conducted inside a nitrogen-filled glove box with oxygen and moisture levels maintained below 1 ppm.

For $MAPbBr_3$ single crystals used for ellipsometric experiments, the precursor MABr (0.748 g) was dissolved in anhydrous dimethylformamide (4 mL) in a 20 mL glass vial to form a clear solution. Then, $PbBr_2$ (2.452 g) was added into the glass vial with stirring to obtain a nearly saturated clear $MAPbBr_3$ solution. The glass vial was then placed onto a hotplate at 50 °C without disturbance for slow evaporation. Bulk $MAPbBr_3$ single crystals with dimensions in the centimeter range can be obtained from the solution after 12 h. These procedures were all performed inside a fume hood.

Transient spectroscopic measurements. The experiments were performed using two different setups: one for broadband visible probe and the other for broadband deep-UV probe.

(a) For the broadband visible probe setup, a 1 kHz regenerative amplifier provides 30 fs pulses at 800 nm with an energy of approximately 720 μJ per pulse. A noncollinear optical parametric amplifier (NOPA) was utilized to generate tunable visible pump pulses with ~15 nm bandwidth and energy ranging from 2–4 μJ per pulse. The probe beam was focused onto a CaF_2 plane to generate white light in the range of 450–750 nm (1.65–2.75 eV).

(b) For the broadband deep-UV probe setup[1,2], a 20 kHz Ti:sapphire regenerative amplifier (KMLabs, Wyvern500), providing 50 fs pulses at 800 nm with an energy of 0.6 mJ. These pulses were used to pumps a NOPA, generating sub-90 fs visible pulses at 13 μJ per pulse in the range of 510–740 nm (1.68–2.43 eV). About 40% of the NOPA output was used to generate broadband UV probe pulses with a bandwidth of ~100 nm through an achromatic doubling scheme[3]. The probe pulses were further compressed using chirp mirrors to achieve <20 fs pulse duration. The relative polarization between the pump and probe beams was set at the magic angle (54.74°) using a half-wave plate to avoid photo-selection effects. After passing through the sample, the transmitted broadband probe beam was focused into a 5 m multi-mode optical fiber, which was coupled to the entrance slit of a 0.25 m imaging spectrograph (Chromex 250is). The beam was dispersed by a 150 gr/mm holographic grating and imaged onto a multichannel detector consisting of a 512-pixel CMOS linear sensor (Hamamatsu S11105) with a pixel size of 12.5×250 μm. The pixel readout rate could reach up to 50 MHz. The typical spot sizes of the pump and probe beams were approximately 120 μm and 50 μm full widths at half-maximum, respectively.

In all measurements, the pump fluence at 400 nm (3.1 eV) was approximately 50 μJ/cm^2 with ~10% uncertainty due to laser power measurement and laser beam spot size. The pump power was

recorded on a shot-to-shot basis using a calibrated photodiode for each pump wavelength, enabling the normalization of the data for the pump power. The thin film perovskite samples were mounted in a film sample holder with a nitrogen gas flow to protect the sample surface. The probe signal was measured after transmission through the sample, and its detection was synchronized with the laser repetition rate.

Temperature-dependent *in-situ* spectroscopic ellipsometry. Temperature-dependent *in-situ* spectroscopic ellipsometry was performed using an M-2000 DI device (J. A. Woollam, USA), which operated in the 193–1690 nm wavelength range, coupled with an INSTEC heating stage, which allowed thermal scanning from room temperature up to 560 °C under an N_2 gas flow. The sample was measured at a minimum of three angles of incidences (65°, 70°, and 75°), at temperatures ranging from 298 to 418 K, which are below the phase transition temperature of ~530 K[4]. The VASE data was fitted using an isotropic "B-spline" mode[5], allowing for the determination of the absorption coefficients and refractive indexes at each measured temperature, and the data analysis was performed using the Complete EASE 6.51 software package. The raw and fitted data are shown in Fig. S8.

DFT calculations. We performed the DFT calculations using the projector-augmented wave method implemented in the Vienna Ab Initio Simulation Package code[6,7]. The GGA and PBE exchange-correlation functional were used, and van der Waals interactions were also included in the calculations using the zero-damping DFT-D3 method of Grimme. A uniform grid of $6 \times 6 \times 6$ k-mesh in the Brillouin zone was employed to optimize the crystal structure of cubic-phase $MAPbBr_3$. The energy cutoffs of the wave functions were set to 500 eV for bulk $MAPbBr_3$. The atomic positions of all the structures were fully relaxed until the Hellman–Feynman forces on each atom were less than 0.01 eV/Å. To assess the influence of the hot-lattice effect on the electronic

structure, we systematically vary the lattice parameters by increments of 0.5%, 1.0%, 1.5%, and 2.0%, thereby emulating the crystal structures at different temperatures.

Supplementary Note S1: The band assignments in the mid-to-deep-UV probe region

The transient spectral traces of $MAPbBr_3$, probed in the UV range with a pump photon energy of 3.1 eV, are presented in Figure 1f. The spectra exhibit three distinct negative peaks located at approximately 3.4 eV, 3.8 eV, and 4.45 eV, accompanied by a positive signal around 4.15 eV. The energies of these bleaching (negative) signals are consistent with the ellipsometric measurements conducted within the same spectral range. Following a similar methodology used for the assignment of interband transitions in $MAPbI_3$,[8,9] we determined the energy distances at each symmetry point in the Brillouin zone (BZ). Accordingly, the ~3.4 eV peak is assigned to the transition from VB3 to CB1 at the R point, while the ~3.8 eV and ~4.45 eV peaks are assigned to transitions between VB1 and CB1 at the M and X points, respectively (Figure S2).

When excited below the energy gap at the M and X points, direct population at these high-symmetry points is not involved. Consequently, the transient signal at ~3.4 eV (R point) exhibits a different sensitivity compared to the bleaches at ~3.8 eV (M point) and ~4.45 eV (X point). This distinction is evident from the early time traces shown in Fig. S6, where the transient signals at ~3.8 eV and ~4.45 eV experience a prompt rise, while the signal at ~3.4 eV rises more gradually, due to the cooling of electrons towards the bottom of CB at the M point. Furthermore, the significant red-shifted peak energy at ~3.8 eV observed at longer delay times indicates that this bleach could be primarily attributed to Coulomb screening rather than band filling (See normalized spectral traces in Figure S23). Conversely, the transient signal at ~4.45 eV shows minimal peak shift, which can be attributed to the relatively flat VB at the Γ-to-X valleys in the BZ.

Supplementary Note S2: The difference between the transient absorption and reflection

In TA measurements, the ΔA signal mainly depends on the ratio of the intensity of transmitted probe light with and without pump excitation, assuming that the loss of transmitted probe light is solely resulting from the sample absorption (assuming that the TA signal is proportional to the change in absorption, ΔT/T∝ΔA[10]).

$$\Delta\mathrm{A} = A_{on} - A_{off} \approx lg\frac{I_0}{I_{out-on}} - lg\frac{I_0}{I_{out-off}} = -lg\frac{I_{out-on}}{I_{out-off}}$$

Where I_0 represents the intensity of incident probe light, I_{out} represents the intensity of the transmitted probe light, A_{on} (or A_{off}) represents the optical density of the samples with (or without) the excitation of pump light, and I_{out-on} (or $I_{out-off}$) represent the intensity of transmitted probe light with (or without) the excitation of pump light.

In transient reflection (TR) measurements, which share the same experimental setup, the TR signal (ΔR/R) can be determined by the ratio of the intensity of reflected probe light with and without pump excitation. However, unlike TA measurements that primarily probe the bulk property of the samples, the TR signal mainly detects variations in photo-induced reflection due to the changes in the refractive index at the sample surface[11,12].

$$\Delta\mathrm{R} = -lg\frac{I_{R-on}}{I_{R-off}}$$

And,

$$\mathrm{R} = \frac{(n-1)^2 + k^2}{(n+1)^2 + k^2}$$

Where n and k represent the real and imaginary parts, respectively, of the complex refractive index ñ=n+ik. Carrier-induced optical effects cause small changes in reflection coefficient R. Therefore, the change in reflectance, ΔR, can be described in terms of the complex index of refraction. Since ΔR is very small, it can be approximately expressed in a linear form by considering Δn and Δk as:

$$\Delta \mathrm{R} = \frac{\partial R}{\partial n}\Delta n + \frac{\partial R}{\partial k}\Delta k$$

The relation between Δn and the Δk is essential for interpreting the signal of the differential reflectivity. Assuming that the magnitude of n is significantly larger than k, the photo-induced ΔR is dominated by Δn, rather than Δk. This is in contrast to photo-induced ΔA, which is predominantly influenced by Δk. Therefore, the TR spectral characteristics and kinetics can differ significantly different from those of TA, even examining the same sample.[10,12–14]

Supplementary Note S3: Estimation of lattice temperature increase in photo-induced experiments

Here we estimate the temperature increase due to photoexcitation of the $MAPbBr_3$ sample in our transient experiments. According to the typical heat absorption equation:[15]

$$\eta \cdot Q = m \cdot C_p \cdot \Delta T$$

Where, Q is the heat absorbed by the material, m is the mass of the heating absorbing subject, C_p is the specific capacity and ΔT is the change in temperature. We assume half of the absorbed photon energy are converted in to heat ($\eta \approx 50\%$). In $MAPbBr_3$, Cp = 8.2 J/(mol·K), ρ = 3.83 g/cm^3, and $m = \rho \cdot l \cdot \pi R^2$, the laser penetration depth l we roughly take the film thickness of ~80 nm[16], R is the pump laser spot radius of ~60 μm, however, as only the most intense center mostly

cause the heating and here we approximately take half of it ($R \approx 30\mu$m). $Q = \frac{P}{r} \cdot \alpha$, where, P is the pump laser power, r is the repetition rate and α is the absorption coefficient at the used pump wavelength (400 nm, ~0.4). Thus, it can be written as:

$$\Delta T = \frac{P \cdot \alpha \cdot \eta}{r \cdot \rho \cdot l \cdot \pi \cdot R^2 \cdot C_p} = \frac{55\mu W \cdot 0.4 \cdot 50\%}{10000Hz \cdot 3.83 {}^{g}/_{cm^3} \cdot 60\ nm \cdot \pi \cdot (30\ \mu m)^2 \cdot 8.2 {}^{J}/_{mol \cdot K}} \approx 96\ K$$

Therefore, for the TDA spectrum, we select the temperature roughly at 373 K (≈273+96=369 K), although all of the TDA spectra exhibit similar profiles.

Supplementary Note S4: The dielectric anomalies below the bandgap

The dielectric anomalies are particularly obvious below the BG at around 2.25 eV. The absorption data presented in our study were derived by fitting the measured spectroscopic ellipsometric data. The fitting process was performed using an isotropic "B-spline" model that simultaneously fits all three sets of measured data, corresponding to different angles of 65°, 70°, and 75°. Therefore, the quality of the data is heavily reliant on the accuracy of the fitting procedure (as shown in Figure S8).

Although the overall fitting appears satisfactory with a mean squared error (MSE) of 2.207, the onset of the "Delta" feature, indicated by the green curve (in Figure S8, the dots are the experimental data, and the solid lines are the fits), poses challenges for accurate fitting. This difficulty arises due to the sharp drop in intensities occurring at approximately 2.3 eV, where the values approach zero. To obtain the best possible fitting results, we employed the "node spacing spectral ranges" method with spectral resolution as high as 0.01 eV within the spectral range of

1.70 to 2.48 eV. Despite these efforts, the fitting-induced anomalies primarily manifest below the bandgap. Therefore, in the temperature-induced differential absorption (TDA) spectra, the energy range was truncated at 2.25 eV. We want to emphasize that these fluctuations are fitting-related artifacts and do not invalidate the key findings and conclusions presented in our study.

Supplementary Note S5: Estimate the timescale for thermal transport

Photo-induced charge carriers transfer their energy to lattice, resulting in the creation of a quasi hot-spot within the $MAPbBr_3$ film (see Figure S23). The decay of this spot follows a non-equilibrium process that is both time and space-dependent. By analogizing the flow of energy per unit area per unit time to electric current, thermal conduction can be likened to discharging a capacitor, and the time constant can be defined: $\tau = \mathrm{RC}$, where $\mathrm{R} = \frac{d}{\sigma A}$, $\mathrm{C} = C_M \rho A d$. Here, d represents the transport distance within the thin film, which can be either the film thickness or the grain size, σ denotes the thermal conductivity (σ =0.4 W/mK), A is the area of the hot-spot, ρ stands for the density (ρ =3.83 g/cm^3), and C_M is the specific heat capacity of $MAPbBr_3$ perovskite (C_M =8.2 J/K · mol).[17]

Thus, $\tau = \frac{d}{\sigma A} C_M \rho A d = \frac{C_M \rho}{\sigma} d^2$, which scales proportionally with d^2.

According to the film preparation method, we used an extremely dilute precursor to fabricate ultrathin films (~30-80 nm) allowing UV light to pass through while also absorbing it. It is noteworthy that the polycrystals formed within these ultrathin films are considerably smaller in size compared to films of standard thickness, typically measuring in the range of tens of nm. Within the films, the hot spot intensity exhibits a Gaussian-like spatial distribution, with its spatial breadth

increasing over time until it reaches the grain boundary, where heat diffusion becomes notably less efficient (Figure S24). As a preliminary estimate, considering i) d = 40 nm, the timescale for thermal transport is ~250 ps; ii) d = 50 nm, the timescale for thermal transport is ~400 ps; iii) d = 60 nm, the timescale for thermal transport is ~550 ps. These estimates align reasonably well with the observations made in our experiments.

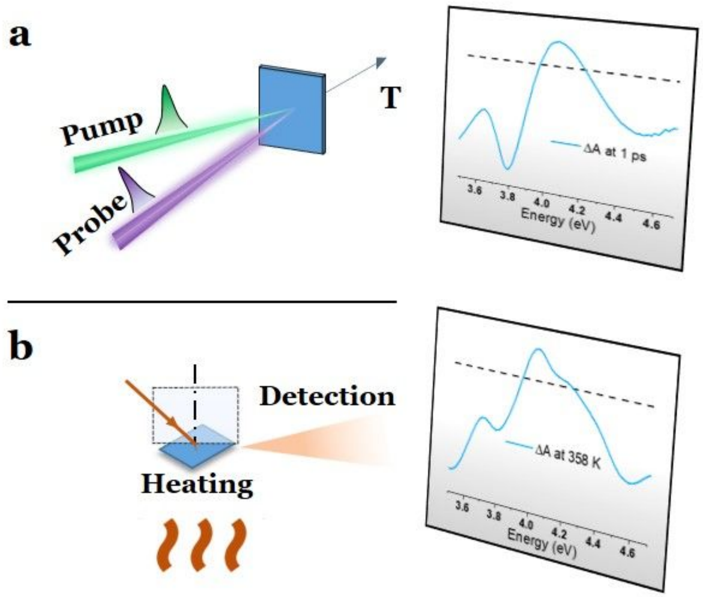

Figure S1. Schematic diagram of photon- and thermally excited differential absorption. **a**, Conventional pump-probe scheme, where the generated ΔOD is determined by the pump photons. **b,** Thermal excitation of the perovskite crystal, where the ΔOD is obtained by subtracting the absorption measured at high temperature with that at room temperature.

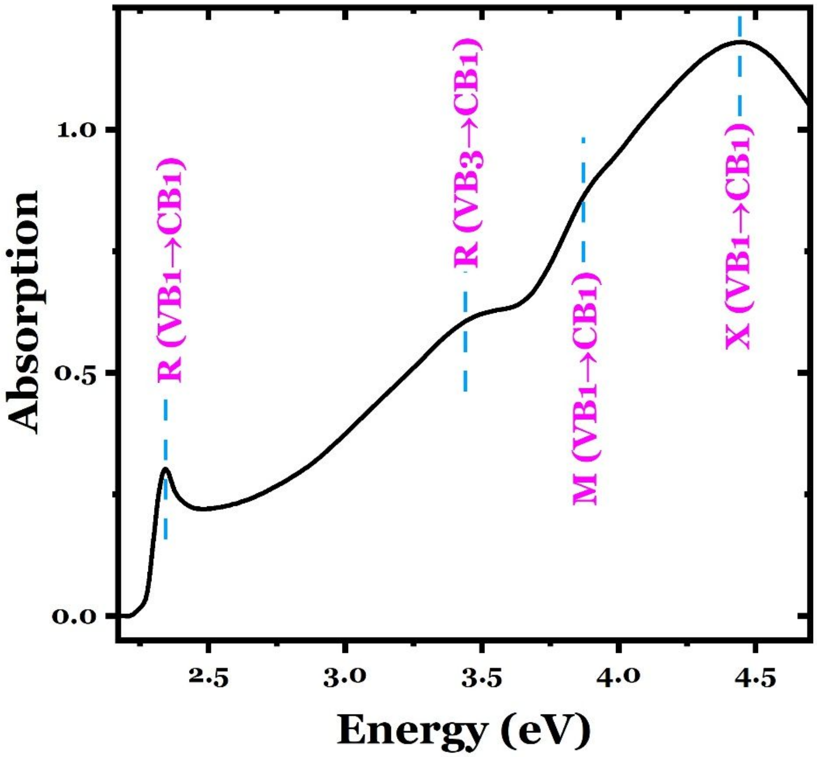

Figure S2. Absorption spectrum of a $MAPbBr_3$ single crystal, highlighting the labeled absorption peaks corresponding to the respective band transitions at various energy levels. The spectrum were obtained by fitting the data measured with spectroscopic ellipsometry.

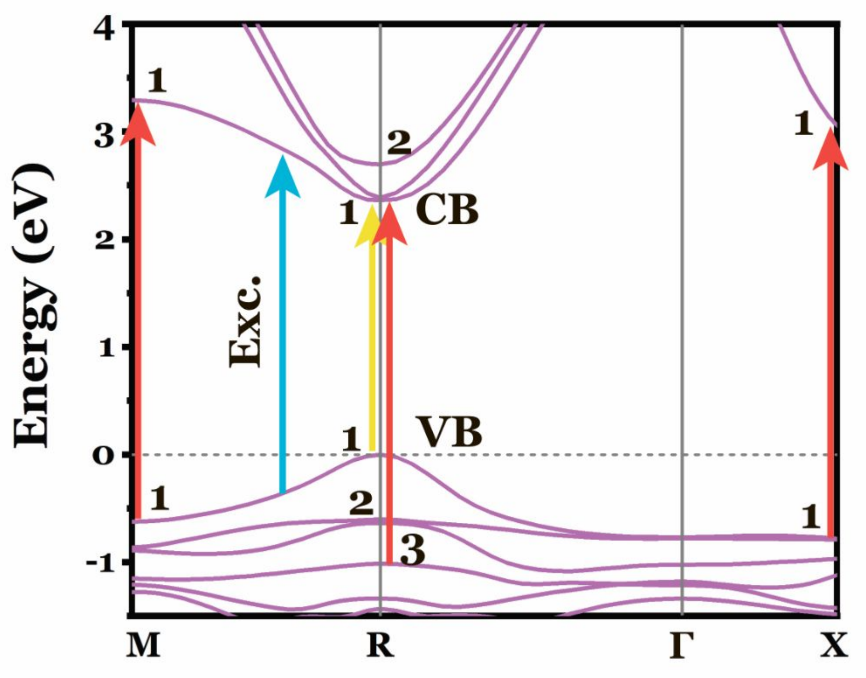

Figure S3. Calculated energy band diagram (the details are described in the method section). The 3.1 eV excitation is indicated by the blue arrow, while the probed bleached signals that can be detected by the employed broadband visible and mid-to-deep-UV probes are represented by the yellow and red arrows, respectively. The number 1, 2 and 3 are labeled in the order of the CB and VB at each symmetry point.

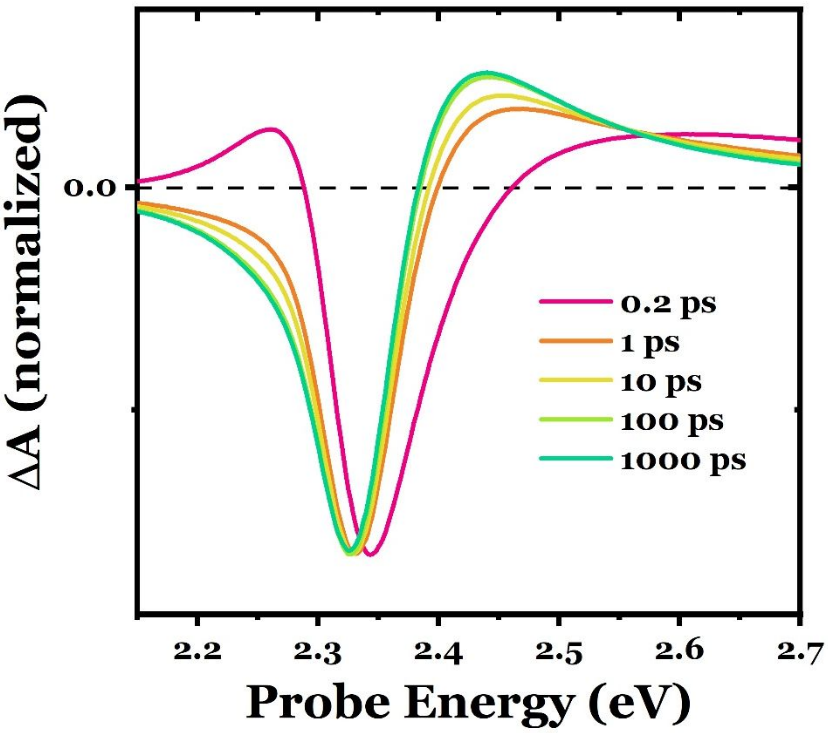

Figure S4. Normalized TA spectral traces at 0.2, 1, 10, 100, and 1000 ps, respectively. The traces are taken from Figure 2b and are normalized at ~2.35 eV.

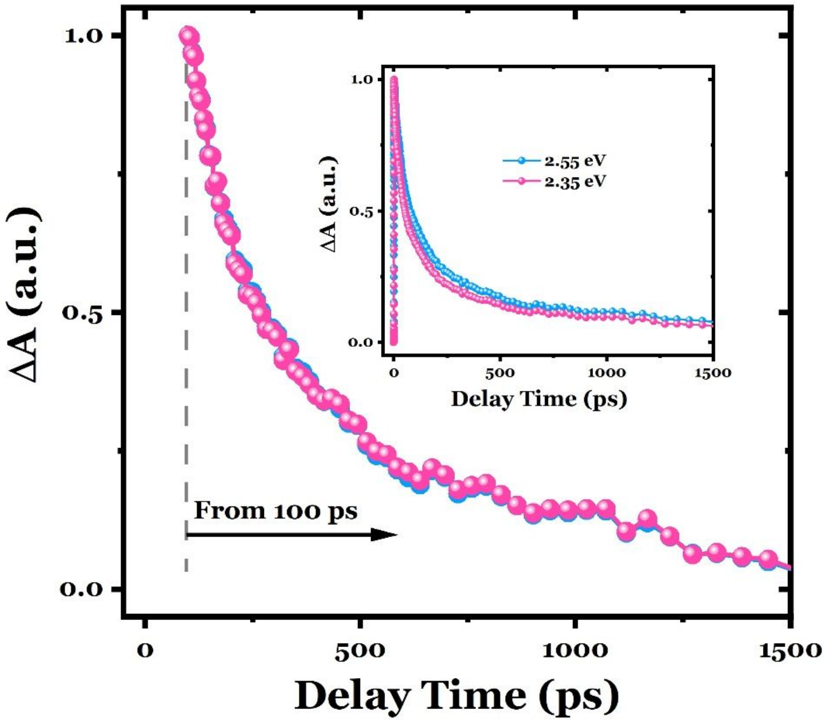

Figure S5. Time traces probed at 2.35 and 4.45 eV, respectively. The traces are cut and normalized at 100 ps to illustrate the long-term decay behaviors. The inset displays a comparison of the normalized full two-time traces.

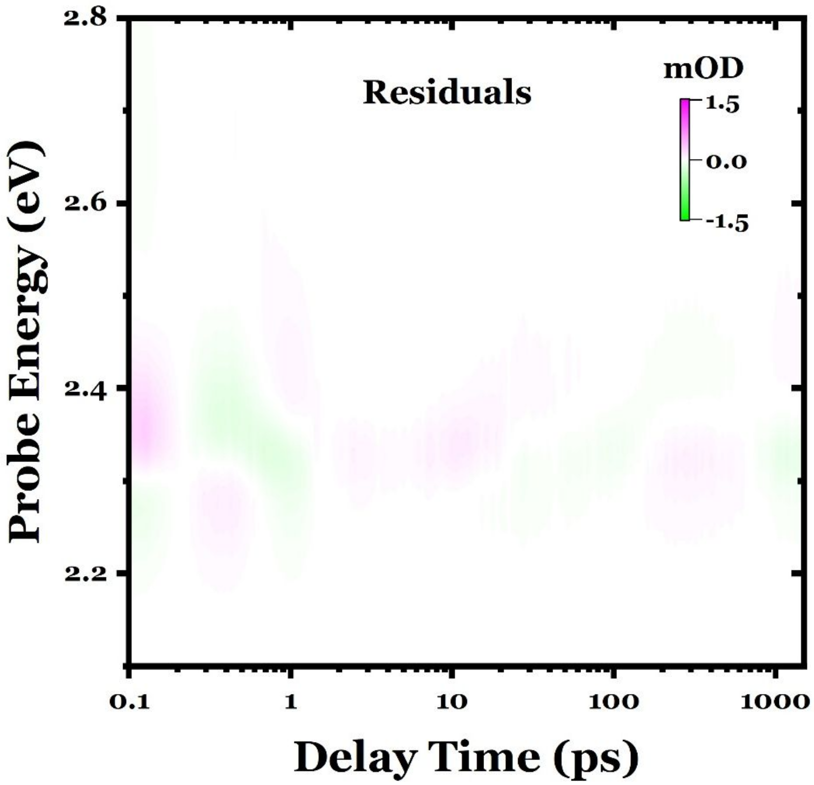

Figure S6. Fitting residuals of the time-energy TA map probed in the visible spectral region. The scale bar is consistent with the experimental and fitted TA maps shown in Fig. 1a and c.

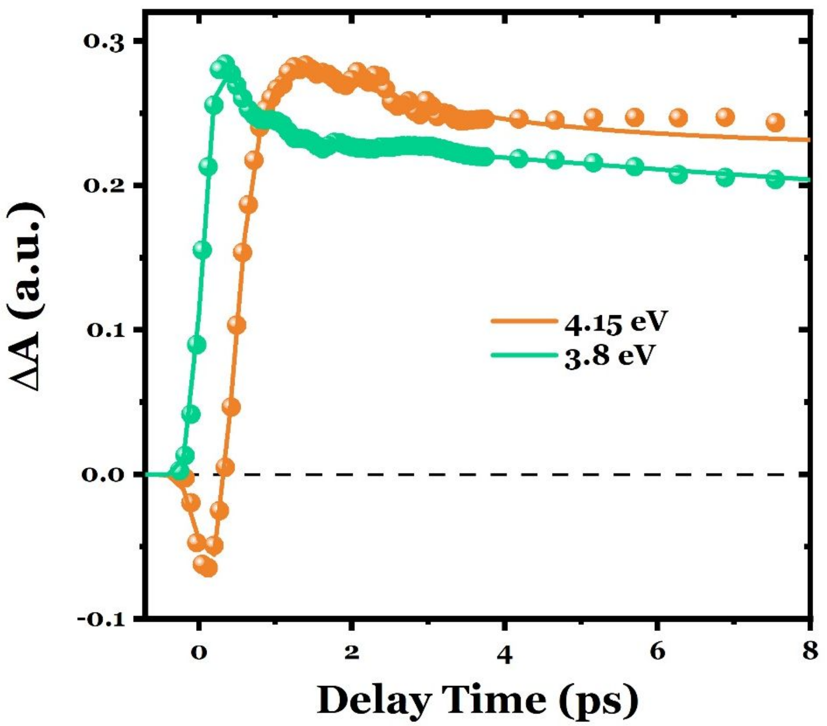

Figure S7. Time traces of the rising TA signals probed at 4.15 eV (positive) and 3.8 eV (negative), respectively. The traces are zoomed in to the first 8 ps and the signals are normalized at their maximum amplitudes. The TA signal at 3.8 eV is flipped to positive for comparison purposes.

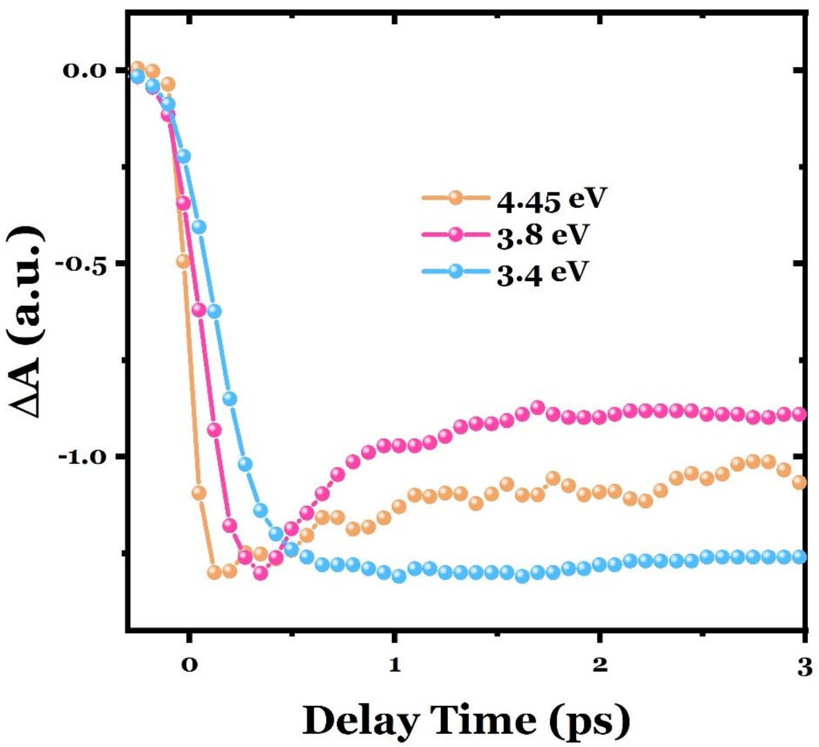

Figure S8. Time traces of the rising TA signals probed at 3.4, 3.8 and 4.45 eV, respectively. The traces are zoomed in to the first 3 ps and normalized at their maximum amplitudes.

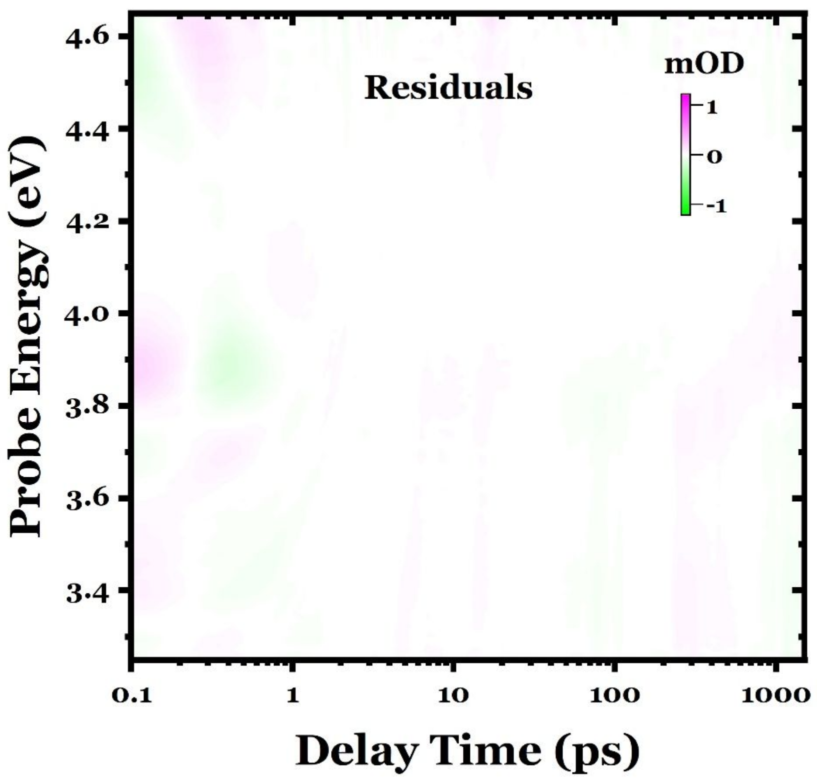

Figure S9. Fitting residuals of the time-energy TA map probed in the UV spectral region. The scale bar is consistent with the experimental and fitted TA maps shown in Fig. 1e and g.

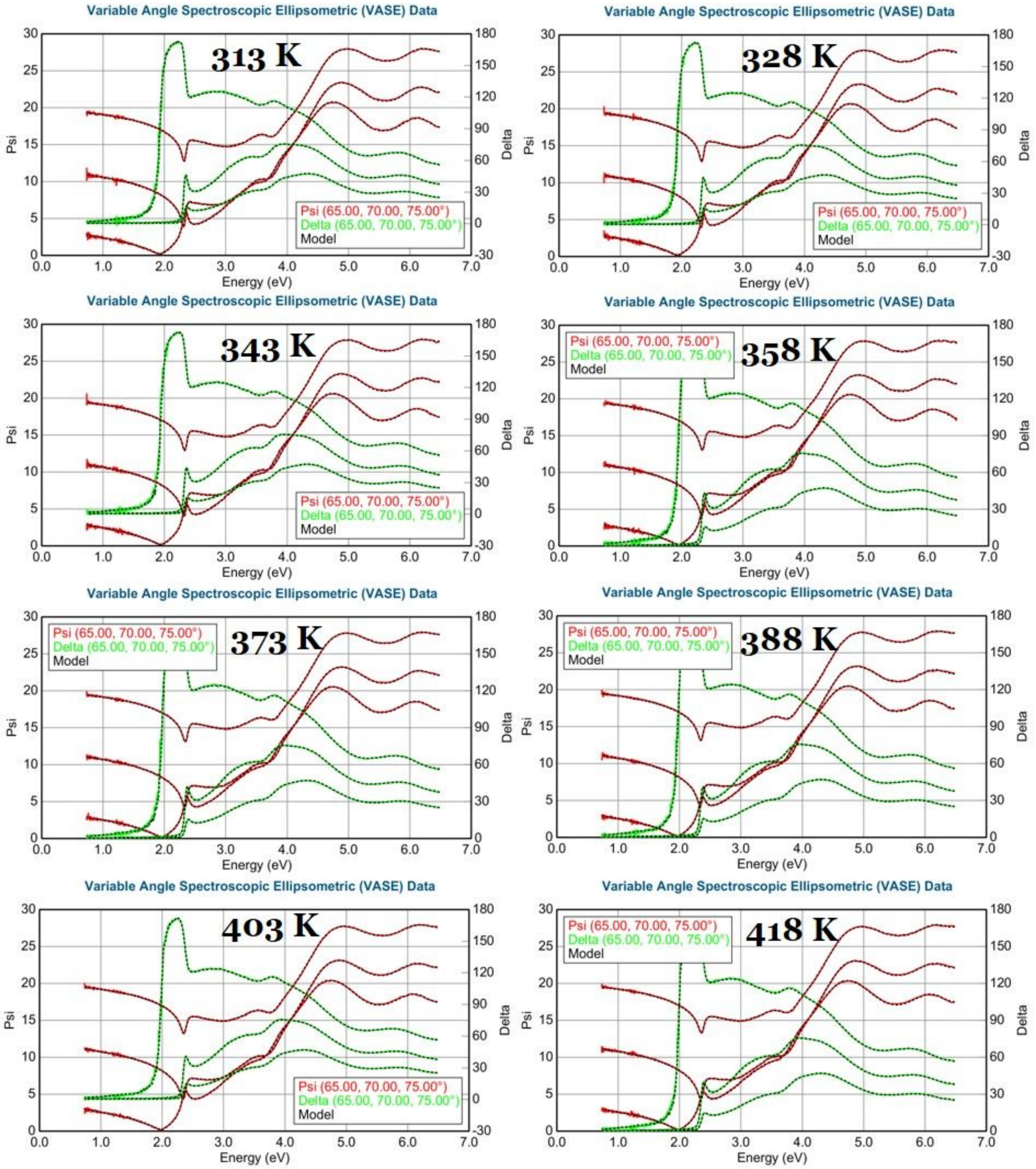

Figure S10. The variable angle spectroscopic ellipsometry (VASE) data of $MAPbBr_3$ perovskite measured at temperatures ranging from room temperature to 418 K in 15 K intervals, with measurements taken at 65°, 70°, and 75°. The dots are the experimental data, and the solid lines are the fits. The absorption coefficients and refractive indexes are extracted based on these fits.

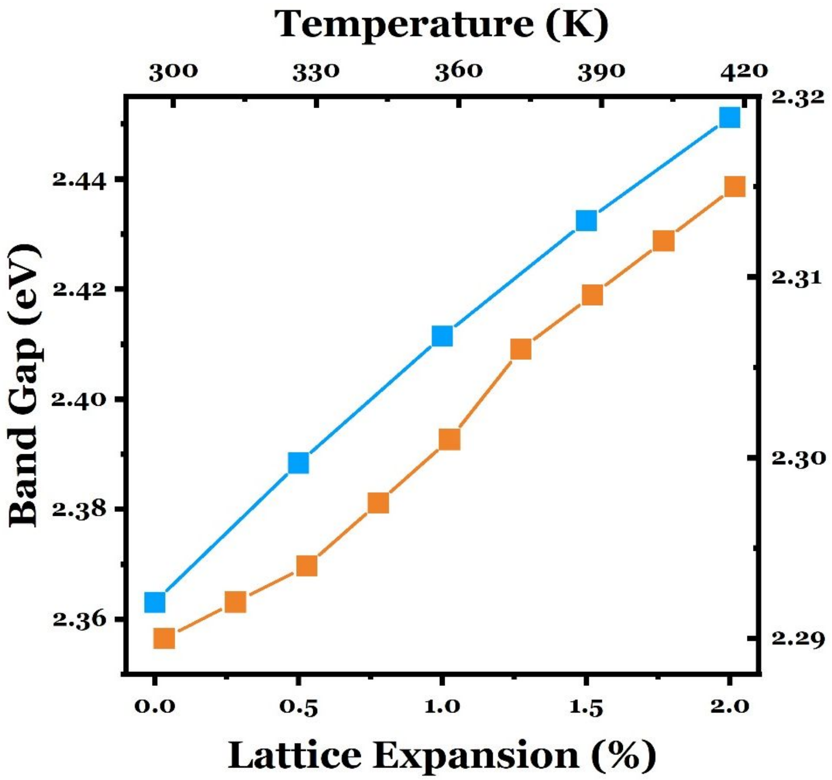

Figure S11. Calculated bandgaps of $MAPbBr_3$ perovskite as a function of lattice expansion, compared to the temperature dependent bandgaps. The experimental band gaps are approximately determined by identifying the points of intersection between the tangent lines to the first absorption peaks and the horizontal coordinate.

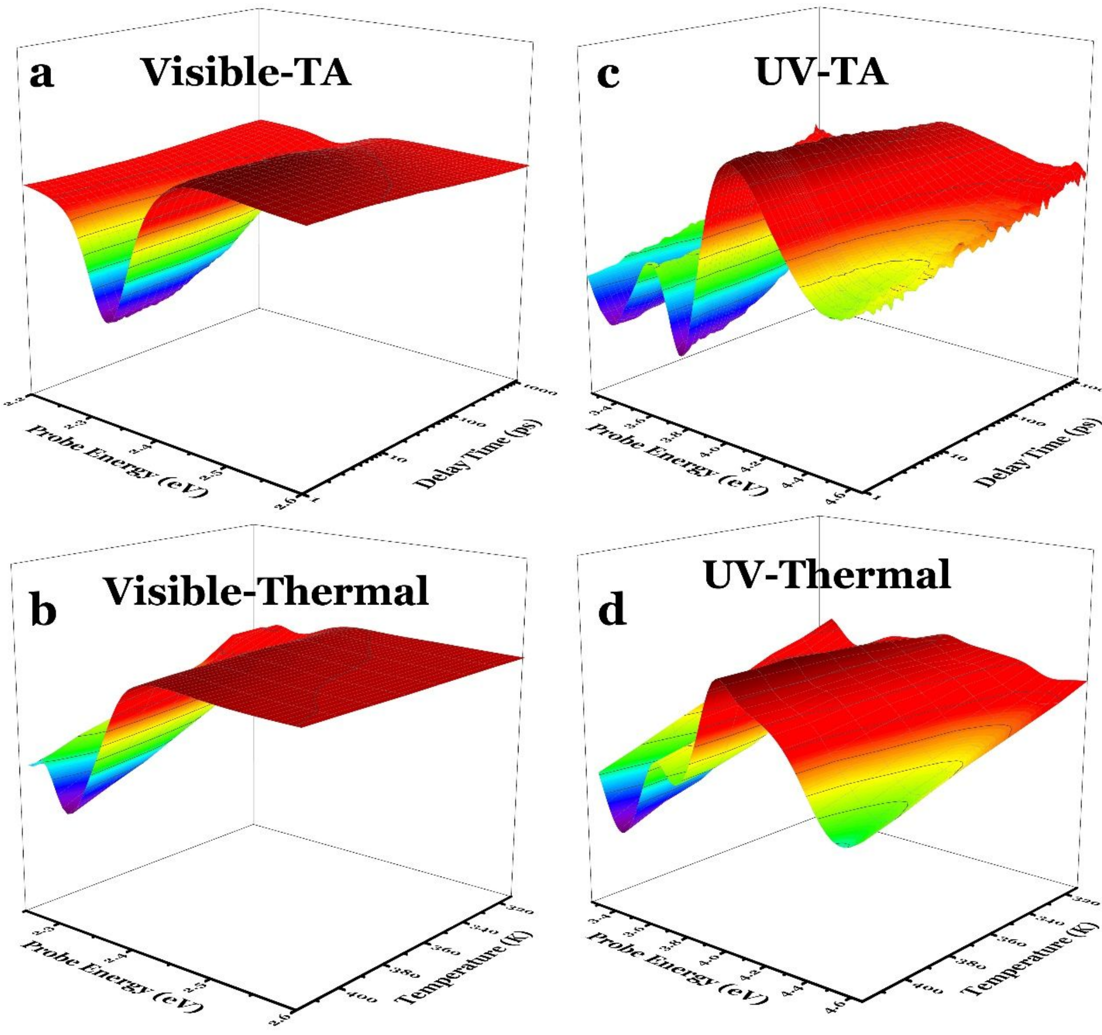

Figure S12. Comparison of photo-induced and thermally induced ΔA profiles for $MAPbBr_3$ perovskite. The TA data for the photo-excited cases were chosen starting from 1 ps, while the thermally excited cases span a temperature range of 313 to 418 K.

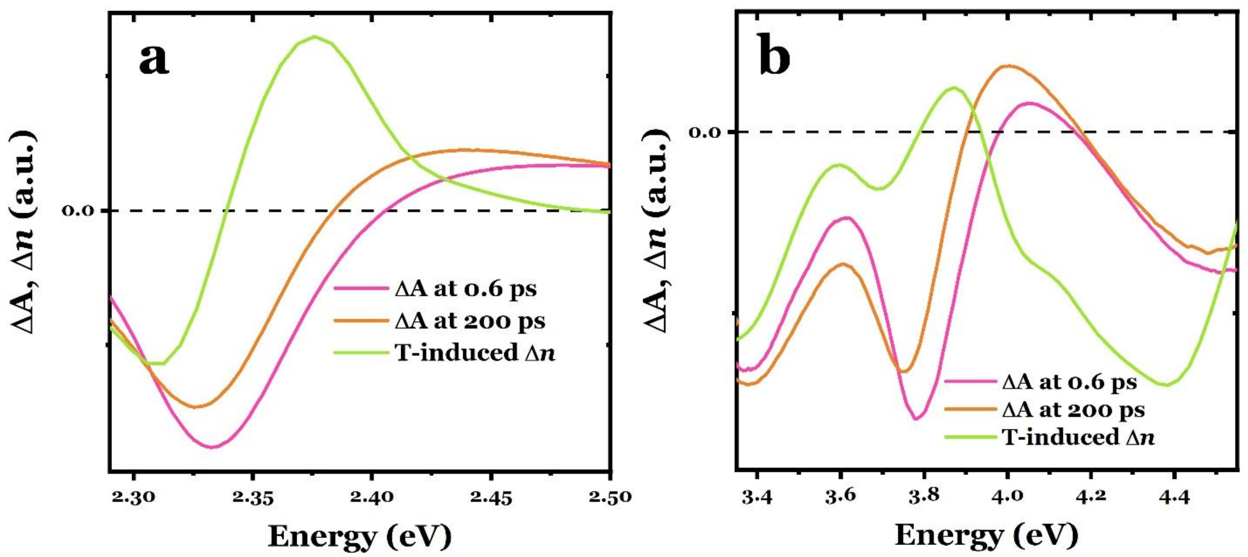

Figure S13. Comparison of photo-induced transients at 0.6 ps, 200 ps and thermal-induced Δ*n* at 373 K in $MAPbBr_3$ perovskite. **a**, Visible spectral region. **b**, Mid-to-deep-UV spectral region.

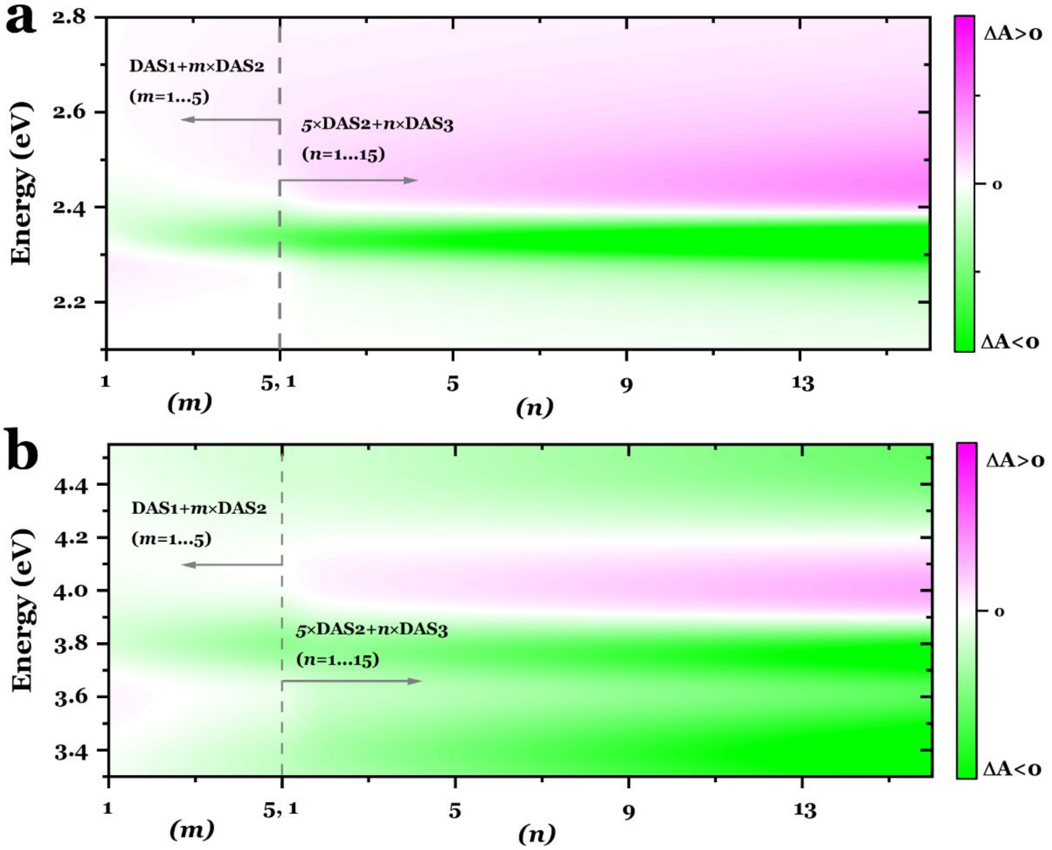

Figure S14. Reconstructed differential absorption map using various ratio of combinations (*m* and *n*). a, Visible spectral region. **b**, UV spectral region. On the left side, the signal amplitude is calculated based on DAS1 + *m*×DAS2 to simulate the complex spectral evolution immediately after photo-excitation. The value of *m* is varied from 1 to 5. On the right side, the signal amplitude is calculated based on 5×DAS2 + *n*×DAS3 to simulate the complex spectral evolution in the long-term component. We vary the value of *n* from 1 to 15.

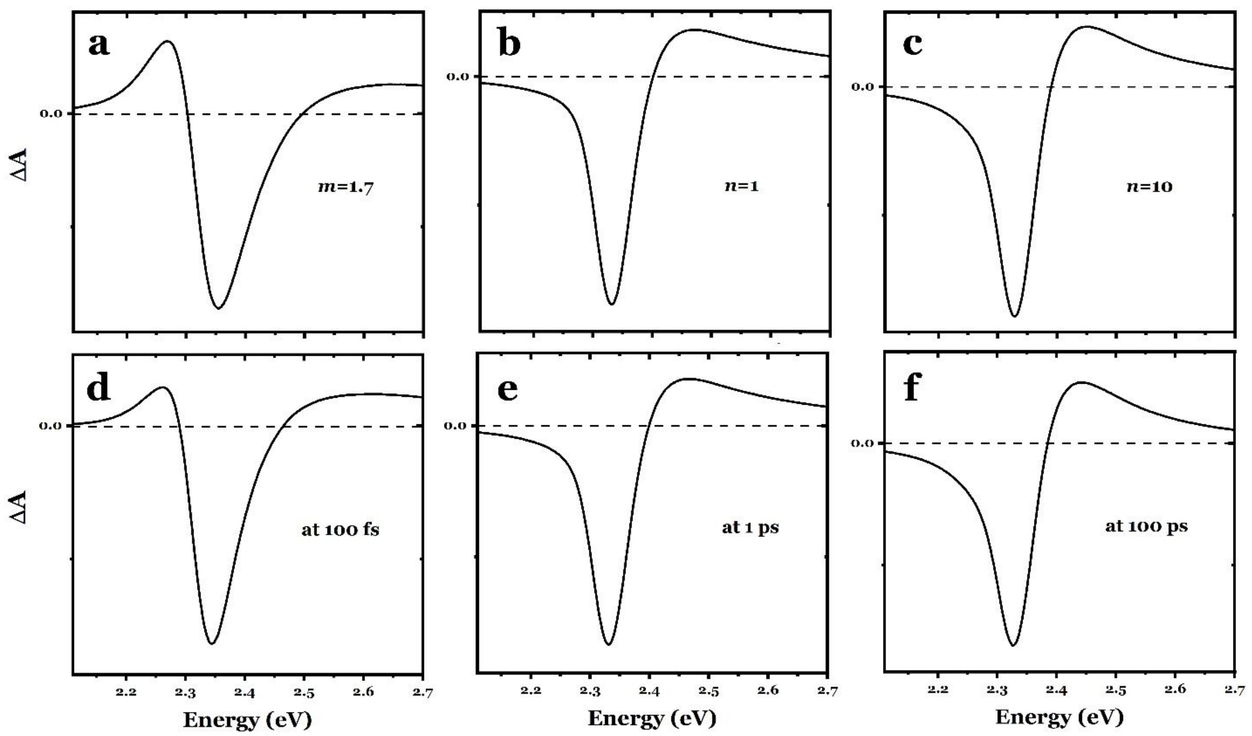

Figure S15. Reconstructed differential absorption spectral profiles in the visible spectral region using various ratio of combinations (*m* and *n*) to simulate the complex spectral evolution immediately after photoexcitation (at 100 fs) and in the long-term component (at 1 ps and 100 ps). The signal amplitude is calculated based on the following: **a,** DAS1 + m×DAS2 (m=1.7). **b,** 5×DAS2 + n×DAS3 (n=1). **c,** 5×DAS2 + n×DAS3 (n=10). The experimental visible-probed spectral traces at **d,** 100 fs. **e,** 1 ps. **f,** 100 ps are also shown.

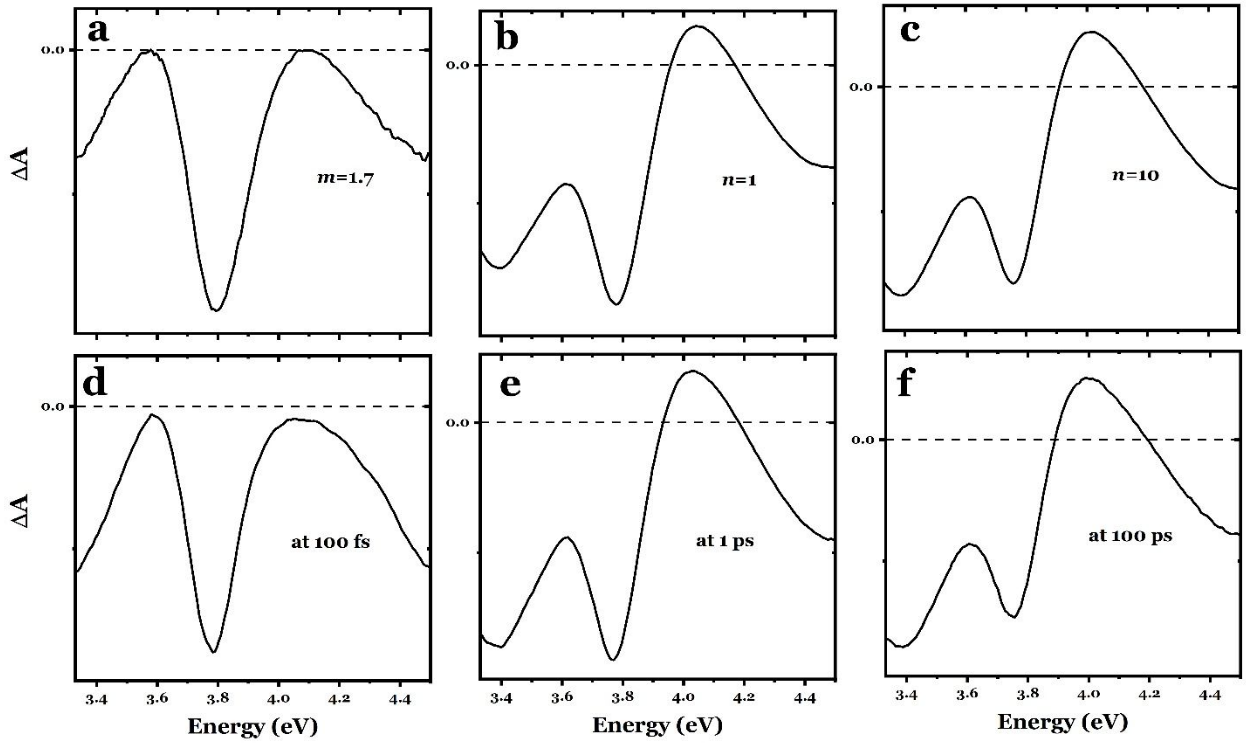

Figure S16. Reconstructed differential absorption spectral profiles in the UV spectral region using various ratio of combinations (*m* and *n*) to simulate the complex spectral evolution immediately after photoexcitation (at 100 fs) and in the long-term component (at 1 ps and 100 ps). The signal amplitude is calculated based on the following: **a,** DAS1 + *m*×DAS2 (m=1.7). **b,** 5×DAS2 + *n*×DAS3 (n=1). **c,** 5×DAS2 + *n*×DAS3 (n=10). The experimental UV-probed spectral traces at **d,** 100 fs. **e,** 1 ps. **f,** 100 ps are also shown.

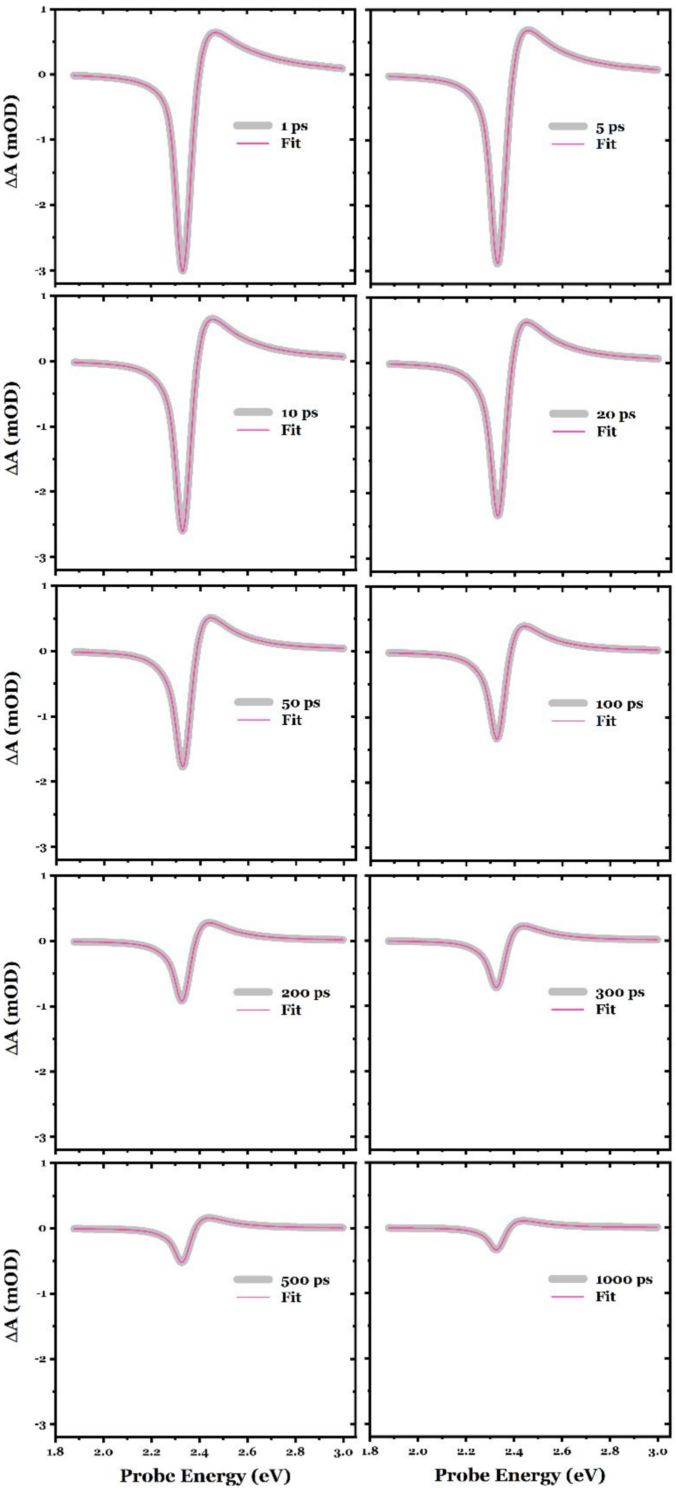

Figure S17. Experimental visible-probed spectral traces and their fittings using different combinations of DAS spectra (a×DAS2 + b×DAS3). The fitting starting from 1 ps, where the band gap renormalization signals have completely vanished. The grey solid lines represent the experimental traces, while the magenta depict the fitted spectra. All figures share the same coordinate system.

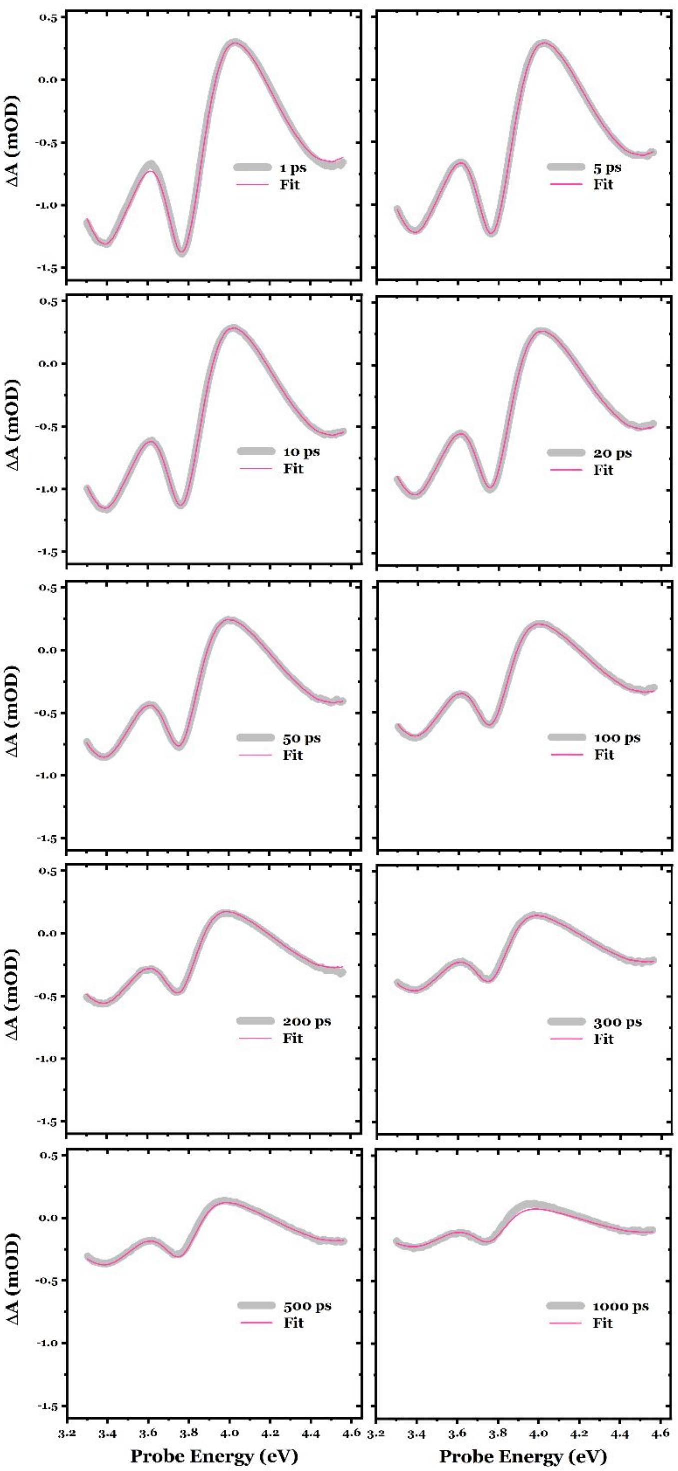

Figure S18. Experimental UV-probed spectral traces and their fittings using different combinations of DAS spectra (a×DAS2 + b×DAS3). The fitting starting from 1 ps. The grey solid lines represent the experimental traces, while the magenta depict the fitted spectra. All figures share the same coordinate system.

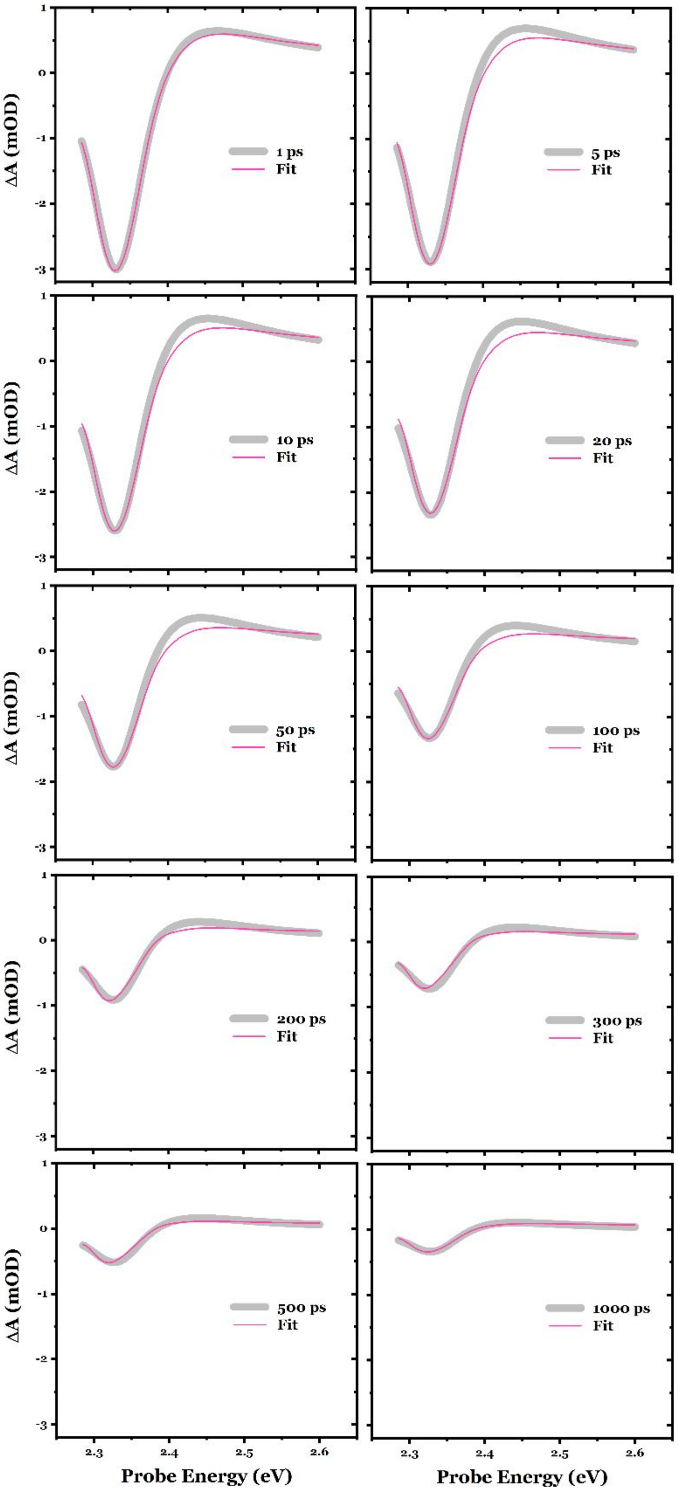

Figure S19. Experimental visible-probed spectral traces and their fittings using combinations of a×DAS2 + b×TDA. The fitting starting from 1 ps, using the same method with Fig. S15. The grey solid lines represent the experimental traces, while the magenta depict the fitted spectra. All figures share the same coordinate system.

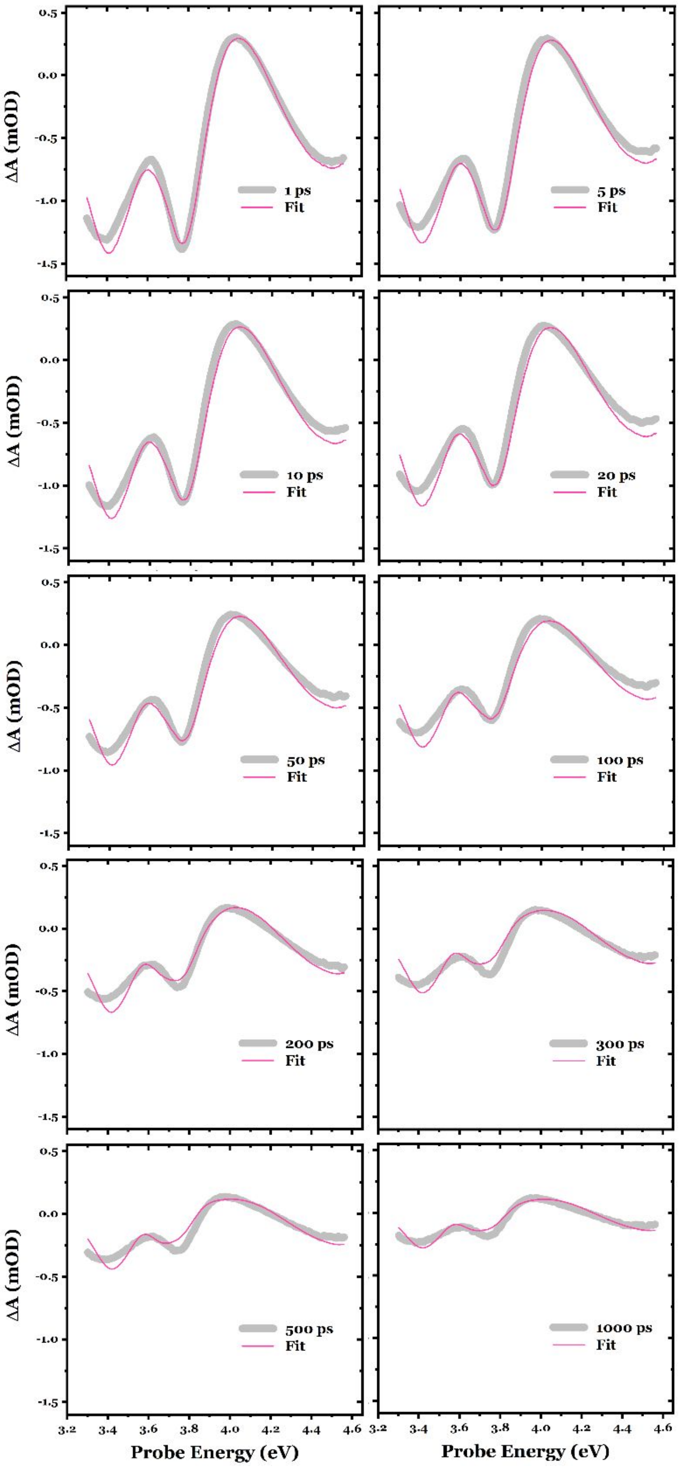

Figure S20. Experimental UV-probed spectral traces and their fittings using combinations of a×DAS2 + b×TDA. The fitting starting from 1 ps, using the same method with Fig. S16. The grey solid lines represent the experimental traces, while the magenta depict the fitted spectra. All figures share the same coordinate system.

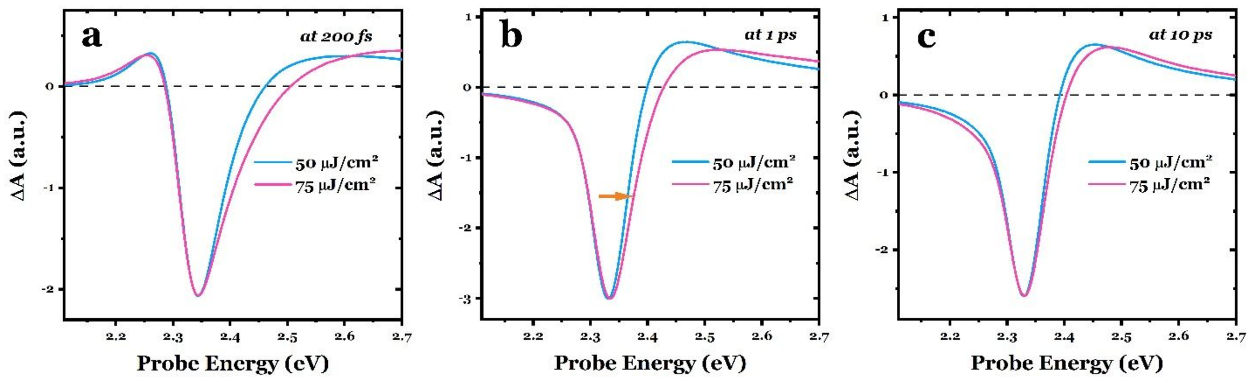

Figure S21. Evolution of visible-probed TA spectral traces under different pump fluences. **a,** at 200 fs. **b,** at 1 ps. **c,** at 10 ps. The blue curves represent the spectral traces used in the main text with a pump fluence of 50 μJ/cm^2, while the red curves represent the spectral traces at the same delay times but with an increased fluence of 75 μJ/cm^2 (1.5 times higher). The two spectral traces are normalized at their signal minimum. Increasing the pump fluence results in immediate broadening of the overall bleaching signal after photo-excitation, particularly on the high-energy side. At a later time delay (1 ps), a ~3.6 meV blue shift in the spectra indicates a larger band gap due to increased lattice heating, consistent with experimental and theoretical results on changes in optical properties induced by temperature. In the long-term component (10 ps), the spectral response under high fluence shows no visible shift, only broadening.

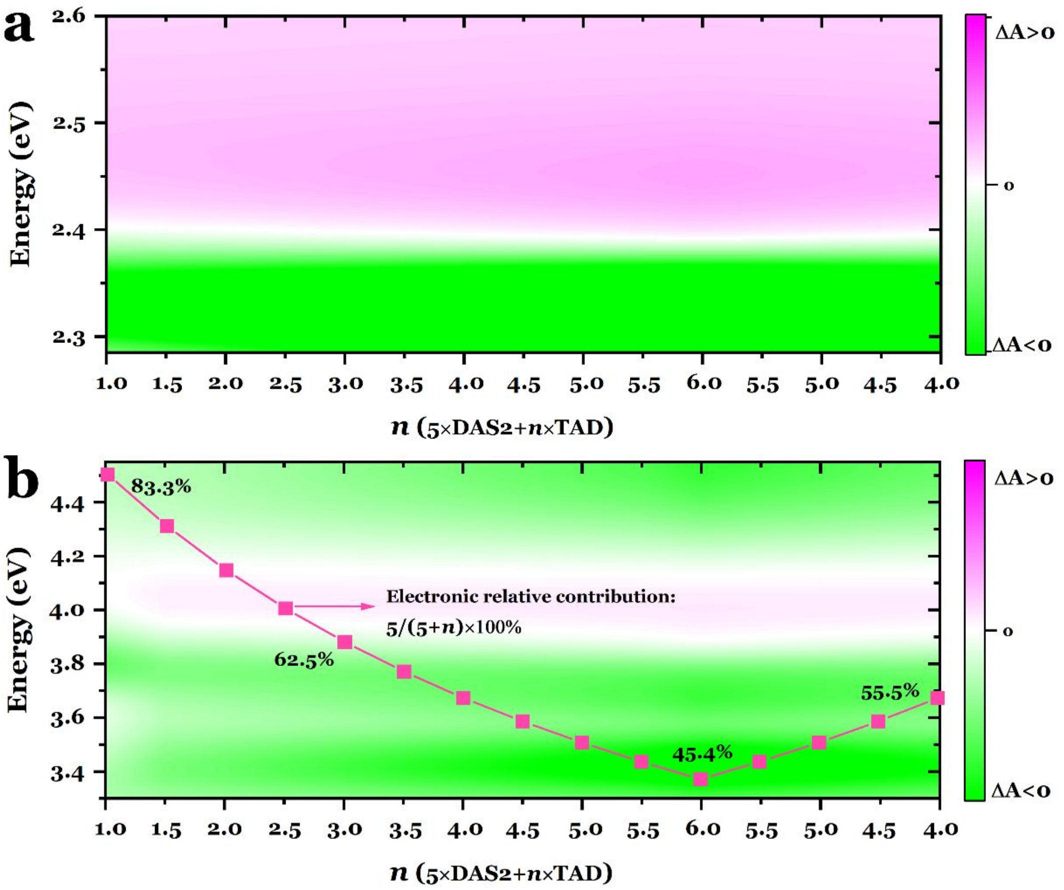

Figure S22. Reconstructed differential absorption map using various ratio of combinations of DAS2 and TDA. **a**, Visible spectral region. **b**, UV spectral region. The signal amplitude is calculated based on 5×DAS2 + n×TDA to simulate the complex spectral evolution in the mid-to-long-term component, i.e., >1 ps. The value of n is varied from 1 to 6, and 6 to 4. It is important to note that, while the relative electronic contribution to the overall TA signal increases at longer delay times, both the electronic and thermal effects are in fact diminishing as a function of delay time.

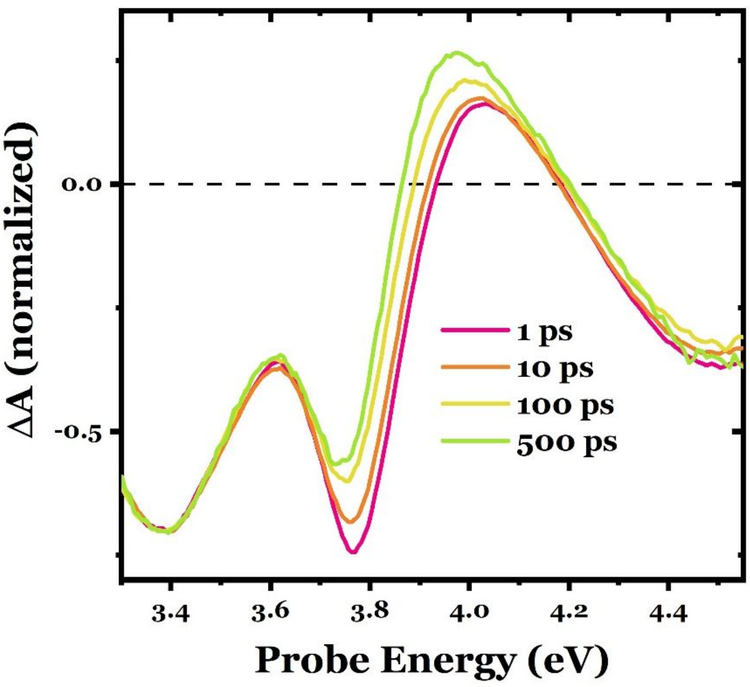

Figure S23. Normalized TA spectral traces at 1, 10, 100, and 500, respectively. The traces are taken from Fig.1f and are normalized at ~3.4 eV.

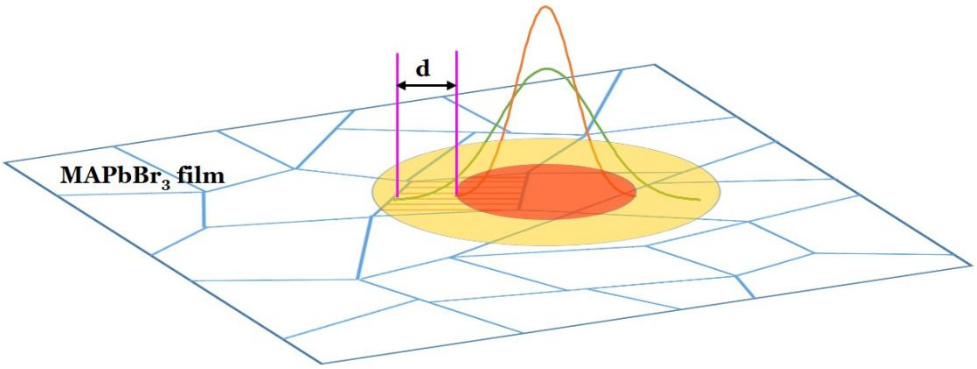

Figure S24. Schematic representation illustrating the thermal decay of a laser-induced hot spot within $MAPbBr_3$ films, depicting heat transfer from the center of the spot in both horizontal and vertical directions.

Reference

(1) Auböck, G.; Consani, C.; Monni, R.; Cannizzo, A.; van Mourik, F.; Chergui, M. Femtosecond Pump/Supercontinuum-Probe Setup with 20 kHz Repetition Rate. *Review of Scientific Instruments* **2012**, *83* (9), 093105.

(2) Auböck, G.; Consani, C.; van Mourik, F.; Chergui, M. Ultrabroadband Femtosecond Two-Dimensional Ultraviolet Transient Absorption. *Optics letters* **2012**, *37* (12), 2337–2339.

(3) Baum, P.; Lochbrunner, S.; Riedle, E. Zero-Additional-Phase SPIDER: Full Characterization of Visible and Sub-20-Fs Ultraviolet Pulses. *Opt. Lett., OL* **2004**, *29* (2), 210–212.

(4) Wang, L.; Wang, H.; Nughays, R.; Ogieglo, W.; Yin, J.; Gutiérrez-Arzaluz, L.; Zhang, X.; Wang, J.; Pinnau, I.; Bakr, O. M. Phonon-Driven Transient Bandgap Renormalization in Perovskite Single Crystals. *Materials Horizons* **2023**.

(5) Johs, B.; Hale, J. S. Dielectric Function Representation by B-Splines. *physica status solidi (a)* **2008**, *205* (4), 715–719.

(6) Kresse, G.; Hafner, J. Ab Initio Molecular Dynamics for Open-Shell Transition Metals. *Physical Review B* **1993**, *48* (17), 13115.

(7) Kresse, G.; Furthmüller, J. Efficient Iterative Schemes for Ab Initio Total-Energy Calculations Using a Plane-Wave Basis Set. *Physical review B* **1996**, *54* (16), 11169.

(8) Leguy, A. M. A.; Azarhoosh, P.; Alonso, M. I.; Campoy-Quiles, M.; Weber, O. J.; Yao, J.; Bryant, D.; Weller, M. T.; Nelson, J.; Walsh, A.; van Schilfgaarde, M.; Barnes, P. R. F. Experimental and Theoretical Optical Properties of Methylammonium Lead Halide Perovskites. *Nanoscale* **2016**, *8* (12), 6317–6327.

(9) Manser, J. S.; Kamat, P. V. Band Filling with Free Charge Carriers in Organometal Halide Perovskites. *Nature Photon* **2014**, *8* (9), 737–743.

(10) Price, M. B.; Butkus, J.; Jellicoe, T. C.; Sadhanala, A.; Briane, A.; Halpert, J. E.; Broch, K.; Hodgkiss, J. M.; Friend, R. H.; Deschler, F. Hot-Carrier Cooling and Photoinduced Refractive Index Changes in Organic–Inorganic Lead Halide Perovskites. *Nature communications* **2015**, *6* (1), 1–8.

(11) Liu, J.; Leng, J.; Wang, S.; Zhang, J.; Jin, S. Artifacts in Transient Absorption Measurements of Perovskite Films Induced by Transient Reflection from Morphological Microstructures. *J. Phys. Chem. Lett.* **2019**, *10* (1), 97–101.

(12) Palmieri, T.; Baldini, E.; Steinhoff, A.; Akrap, A.; Kollár, M.; Horváth, E.; Forró, L.; Jahnke, F.; Chergui, M. Mahan Excitons in Room-Temperature Methylammonium Lead Bromide Perovskites. *Nature communications* **2020**, *11* (1), 1–8.

(13) Chen, X.; Lu, H.; Yang, Y.; Beard, M. C. Excitonic Effects in Methylammonium Lead Halide Perovskites. *J. Phys. Chem. Lett.* **2018**, *9* (10), 2595–2603.

(14) Yang, Y.; Yan, Y.; Yang, M.; Choi, S.; Zhu, K.; Luther, J. M.; Beard, M. C. Low Surface Recombination Velocity in Solution-Grown CH3NH3PbBr3 Perovskite Single Crystal. *Nature communications* **2015**, *6* (1), 1–6.

(15) Hashimoto, S.; Werner, D.; Uwada, T. Studies on the Interaction of Pulsed Lasers with Plasmonic Gold Nanoparticles toward Light Manipulation, Heat Management, and Nanofabrication. *Journal of Photochemistry and Photobiology C: Photochemistry Reviews* **2012**, *13* (1), 28–54.

(16) Brooks, W. S. M.; Irvine, S. J. C.; Barrioz, V. High-Resolution Laser Beam Induced Current Measurements on Cd0. 9Zn0. 1S/CdTe Solar Cells. *Energy Procedia* **2011**, *10*, 232–237.

(17) Jung, Y.; Lee, W.; Han, S.; Kim, B.-S.; Yoo, S.-J.; Jang, H. Thermal Transport Properties of Phonons in Halide Perovskites. *Advanced Materials* **2023**, 2204872.
